# Moonlight drives the energy balance and annual cycle of a nocturnal forager

**DOI:** 10.1126/sciadv.aed8204

**Published:** 2026-05-01

**Authors:** Carlos Camacho, Gabriel Norevik, Pedro Sáez-Gómez, Paula Hidalgo-Rodríguez, Julio Rabadán-González, Susanne Åkesson, Anders Hedenström

**Affiliations:** ^1^ Department of Ecology and Evolution, Estación Biológica de Doñana (EBD-CSIC), Sevilla 41092, Spain.; ^2^ Department of Biology, Centre for Animal Movement Research (CAnMove), Lund University, Lund 221 00, Sweden.; ^3^ Department of Molecular Biology and Biochemical Engineering, Universidad Pablo de Olavide, Sevilla 41013, Spain.; ^4^ Observation.org, Sevilla 41111, Spain.

## Abstract

Behavioral, physiological, and life-history adaptations to the lunar cycle are global phenomena across trophic levels and ecosystems yet remain poorly understood because of the challenges of studying free-living organisms at night. We show that the lunar cycle regulates both daily foraging activity and foraging success in the red-necked nightjar—a nocturnal avian insectivore—and that moonless periods lead to energy deficits that trigger synchronized energy-conservation responses. These cyclical imbalances cascade into fluctuations in fuel reserves and influence the timing of key annual life-history events, including migration and reproduction, but not molt. Despite adaptations to offset lunar constraints, nightjars’ annual cycle remains governed by the moon’s monthly rhythm, underscoring its pervasive influence on nocturnal life.

## Teaser

Moonlight shapes foraging success, energy balance, and timing of annual life-history events in a nocturnal bird.

## INTRODUCTION

Most life forms are exposed to periodic environmental changes across multiple timescales arising from the translational and rotational motion of celestial bodies, promoting adaptations to geophysical cycles (*1*–*3*). These adaptations include, for instance, seasonal migrations in pursuit of resources for reproduction and survival (*2*, *4*), adjustments of digestive performance to maximize energy intake during resource peaks (*5*, *6*), and controlled reductions in body temperature and metabolic rate to conserve energy during fasting (*7*, *8*). For nocturnal organisms, the lunar cycle imposes a recurring pattern of nocturnal illumination that shapes behavior, physiology, and life-history traits. Moonlight-associated responses have been documented across trophic levels in aerial, terrestrial, and aquatic ecosystems, demonstrating that the lunar cycle can influence ecological processes on a global scale (*9*–*12*). Despite this, the effects of the lunar cycle on organismal ecology and evolution remain poorly understood because of the inherent challenges of studying free-living organisms at night (*13*).

Nightjars (Caprimulgidae) are a cosmopolitan group of crepuscular and nocturnal aerial insectivores that primarily forage in dim-light conditions and rest motionless during the day (*14*), making them an excellent system to investigate lunar light influences on organismal behavior and physiology (*15*–*17*). Previous studies on several species have reported correlations between the lunar cycle and both daily foraging activity and the timing of energy-demanding annual life-history events, such as breeding and migration (*15*–*17*), suggesting that moonlight may impose periodic constraints on energy acquisition. However, the mechanisms linking lunar illumination to individual activity, energy allocation, and life-history decisions remain poorly understood. Caprimulgids can gorge on insects during brief crepuscular foraging periods and minimize metabolic costs through reversible reductions in body temperature (torpor), strategies that likely enable them to endure the ephemeral foraging opportunities of moonless nights (*7*, *8*, *18*). Together, these traits position nightjars as an excellent model for understanding how lunar cycles shape behavior, physiology, and life-history trade-offs in nocturnal animals.

Here, we investigated red-necked nightjars (*Caprimulgus ruficollis*; hereafter nightjars) to assess how the lunar cycle affects daily foraging behavior and success, how moonlight-related constraints on foraging influence individual energy reserves and the use of energy-saving mechanisms such as torpor, and how these effects scale up to synchronize key annual cycle events, including reproduction, feather molt, and migration (fig. S1). To assess foraging success, energy gain, and temporal allocation of breeding and molt, we used long-term (2011 to 2020) field data collected in Doñana, Spain. To monitor flight activity, torpor occurrence, and migration timing, we equipped 74 adult nightjars with custom miniaturized multisensor data loggers (MDLs) during 2016 to 2020. In addition, five individuals carried Global Positioning System (GPS) loggers that recorded space use in relation to both lunar and annual cycles.

## RESULTS

### Lunar cycle effects on daily foraging activity and food intake

**Fig. 1.**
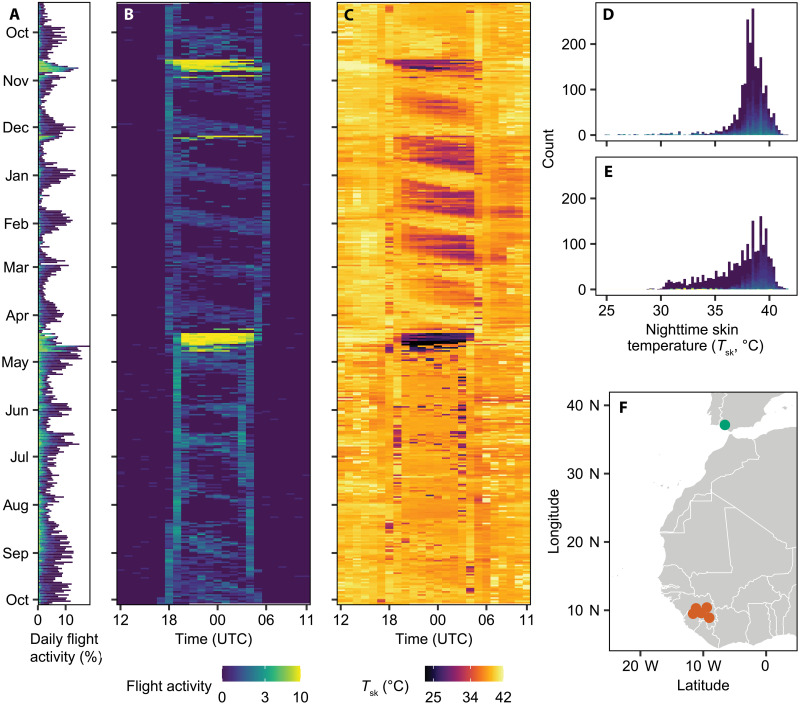
Daily and annual flight activity and temperature dynamics of a red-necked nightjar as recorded by a MDL. (**A**) Percentage of accelerometer registrations per day that have detected flight activity across a full annual cycle (outside migratory flight events). (**B**) Flight activity data from (A) distributed per hour. UTC, universal time coordinated. (**C**) Hourly skin temperature data (truncated to 25° and 42°C to facilitate visualization). Distribution of recorded skin temperatures during breeding (**D**) and nonbreeding (**E**) seasons. Colors in (A), (B), (E), and (F) refer to the average flight activity level per hour (0 to 10). (**F**) Map showing the breeding (study) site (green) and nonbreeding locations (orange) of five GPS-tracked nightjars.

The year-round acceleration data from the MDLs revealed consistent periodic patterns of activity (Fig. 1 and fig. S2), closely matching those reported in other caprimulgids (*15*, *16*). The daily number of flight detections fluctuated across the lunar cycle, peaking at full moon and reaching minima around new moon (Fig. 1A). This periodicity mirrored the intermittent flight activity typical of nightjars’ erratic “fly-catching” foraging tactic (*19*). Foraging primarily occurred in dim light at dusk and dawn and during moonlit nights, the latter producing recurring diagonal bands of nocturnal activity visible in actograms throughout the annual cycle (Fig. 1B) (*17*). The skin temperature of active birds, inferred from externally mounted temperature sensors included in the MDLs, showed little variation around the overall mean of 38°C, comparable to body temperatures typically found in euthermic Caprimulgiformes, indicating that skin temperature provides a reliable proxy for body temperature (Fig. 1, C to E, and table S1) (*20*–*22*). Occasional drops below 35°C occurred during periods of activity in the breeding season but were otherwise mainly associated with inactivity during the nonbreeding period in Africa (Fig. 1, D and E, and fig. S3). The combination of abrupt temperature drops and sustained high activity, indicative of continuous flight at a high altitude, made it easy to distinguish the brief migratory journeys between our Spanish study site and nonbreeding staging areas in sub-Saharan West Africa (Fig. 1, B, C, and F) (*21*).

**Fig. 2.**
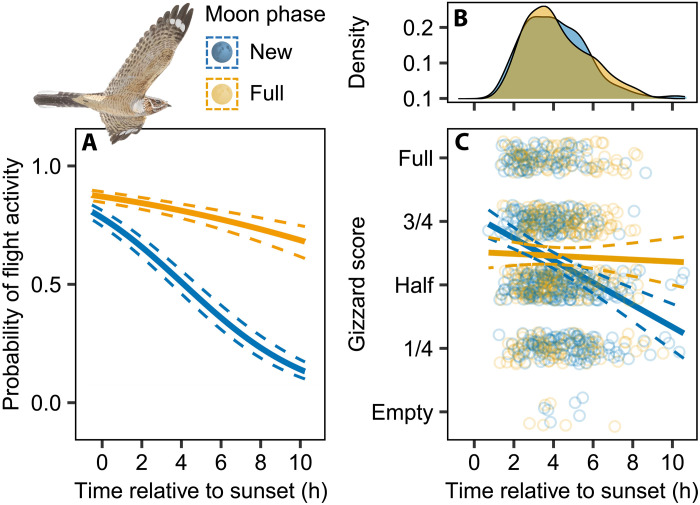
Estimated foraging activity and success of nightjars during the breeding season in relation to sunset in contrasting moon phases. (**A**) Effects of moon phase on flight activity recorded by the MDLs (estimates and 95% confidence intervals). (**B**) Density histogram summarizing daily timings of gizzard records. h, hours. (**C**) Effects of moon phase on gizzard score in nightjars trapped in Doñana (estimates and 95% confidence intervals). Dots represent individual records (with vertical offset to facilitate interpretation). New and full moon phases correspond to periods with <15 and >85% illuminated visible moon disk, respectively. Nightjar illustration: A. Ojea, used with permission.

Focusing on the breeding season, we compared nighttime flight activity and foraging success between nights around the new moon and moonlit nights around the full moon (<15% and >85% of the lunar disk illuminated, respectively; Fig. 2). On dark nights, the probability of activity decreased sharply after sunset, whereas it remained above 60% throughout moonlit nights [generalized linear mixed model (GLMM): *P* < 0.001; Fig. 2A and table S2], indicating that moonlight enables the birds to continue foraging deep into the night. It is important to confirm whether increased foraging activity under moonlight conditions actually improves daily foraging success, given that nightjars might also increase their activity in response to reduced prey accessibility because of moonlight avoidance in insects (*23*). This can be done by quantifying food acquisition across lunar cycles. Caprimulgids can store large amounts of digesta in their gizzard—the muscular stomach of birds (figs. S4 and S5) (*18*, *19*)—making it possible to obtain accurate field estimates of foraging success through noninvasive external palpation and assessment of gizzard fullness (*24*).

Consistent with activity records, gizzard data confirmed a strong positive effect of moonlight on foraging success [linear mixed model (LMM): *P* < 0.001; Fig. 2, B and C, and table S3]. Gizzard fullness decreased throughout dark nights, indicating that feeding ceased after dusk, but remained at intermediate levels throughout moonlit nights (Fig. 2C). The apparent avoidance of full gizzard loads during moonlit nights is expected, as carrying excessive digesta imposes mass-dependent costs on locomotion in aerial organisms (*6*, *25*–*27*). Nevertheless, it indicates a necessity for nightjars to maintain excess food-storage capacity throughout the lunar cycle to cope with sudden foraging constraints, such as those experienced during new moon nights (*5*).

Caprimulgids are substantially efficient foragers, and gizzards crammed full of insects are often recorded in individuals captured within 15 to 20 min after sunset (*18*, *19*). We therefore hypothesize that the maximum storage capacity of the gizzard and food processing rate act as key constraints on the birds’ daily metabolizable energy intake (hereafter, energy intake). On the basis of body mass differences between lean nonbreeding birds with either a full (mean ± SD: 98.86 ± 6.87 g; *n* = 125) or empty gizzard (mean ± SD: 87.14 ± 8.44 g; *n* = 18), the gizzard capacity was estimated at 11.72 g (~13% of lean body mass). This value is lower than the 20 to 25% reported for other caprimulgids (*18*) yet approaches the maximum relative gizzard mass across all migratory birds (*28*). The relatively large size of nightjar gizzards, despite the general selective pressure on aerial organisms to minimize the mass of organs that are not essential for flight (*27*), further underscores the adaptive value of maintaining excess food-storage capacity in these birds. The food processing rate averaged 1.78 g/hour ± 0.39 SE, meaning that it takes ~6.5 hours for the contents of a full gizzard to be emptied into the intestine (table S4). Food processing in birds is generally much faster than this (10 to 100×) (*29*), suggesting that the rate of food processing represents a digestive bottleneck limiting energy intake during moonlit nights (*25*), whereas gizzard capacity sets the maximum potential energy intake during dark nights lasting longer than 6.5 hours.

Lunar effects on energy intake are expected to vary across the nightjar’s distribution range because of latitudinal and seasonal changes in night duration (*30*). To capture the full extent of moonlight effects across the annual cycle, we estimated daily energy intake under contrasting moon phases and seasons. During the breeding season (1 to 30 June, ~9 hours between sunset and sunrise) and assuming constant gizzard capacity and food processing rate and an ad libitum supply of arthropods (~20.9 kJ/g dry mass, equivalent to ~88.4 kJ for a full gizzard; fig. S6) (*28*, *29*), nightjars could increase their energy intake from 176.7 kJ on dark nights (i.e., one “evening gizzard” and one “morning gizzard”) to 210.3 kJ on moonlit nights, representing a 19% increase. During the nonbreeding season, the lunar-cycle effect on energy intake is expected to double because of the longer nights (~12 hours), resulting in a 42% increase to 250.5 kJ.

### Lunar cycle effects on annual energy budget

**Fig. 3.**
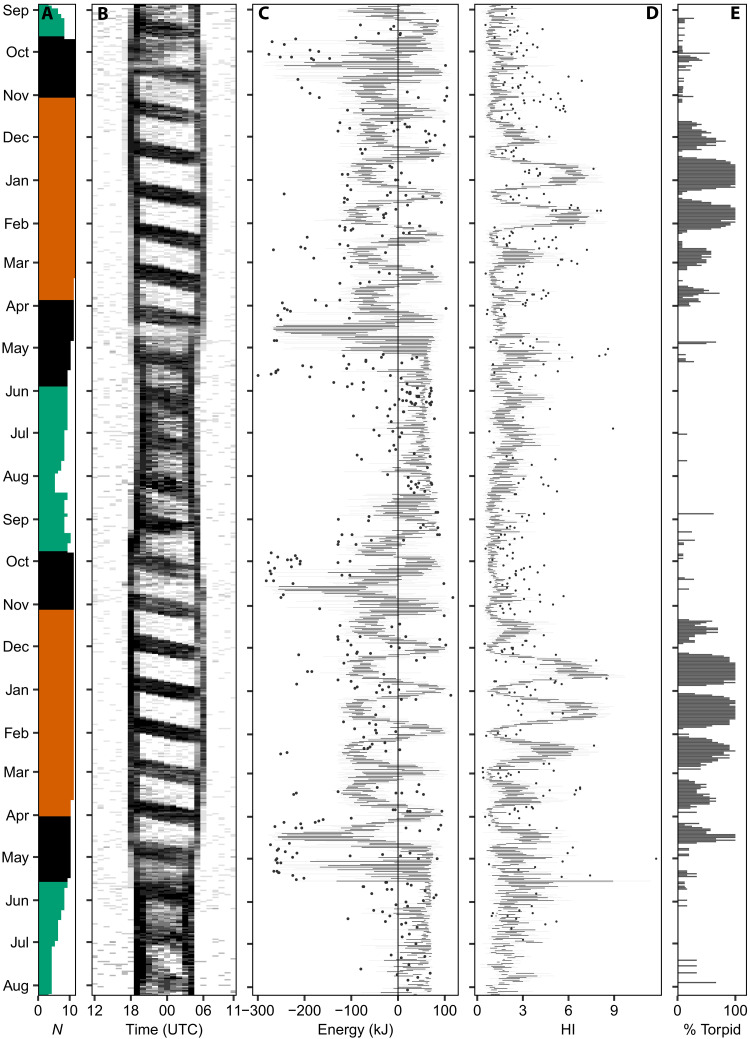
Population-level synchrony of lunar-driven activity, energy balance, and thermoregulation at a daily scale across two annual cycles. (**A**) Bar plot showing the number of individuals tracked and delimitation of breeding (green), nonbreeding (orange), and migration (black). (**B**) Heatmap showing the hourly fraction of active birds from none (white) to all (black). (**C**) Box plot of the distribution of calculated daily energy balance [metabolizable energy intake − (field metabolic rate + locomotion costs proportional to activity records)]. (**D**) Box plot of within-individual variation in nocturnal skin temperatures, expressed as the HI, an index proportional to the magnitude of deviations from an individual’s mean skin temperature. (**E**) Fraction of nightjars recording a drop in skin temperature >2 SD below the mode (~35°C), indicative of shallow torpor.

To understand how endogenous and lunar cycle–associated constraints interact to shape the energy intake dynamics, field metabolic rate, flight costs, and the resulting daily energy balance (hereafter, energy balance) across the annual cycle, we parameterized individual-based models using activity data (Fig. 3, A to C; see Materials and Methods). The temporal pattern of flight activity was consistent across individuals, synchronized with the timing of dusk, dawn, and moonlit nights, particularly during the nonbreeding season (Fig. 3B). Modeled energy balances exhibited clear lunar cycle–driven fluctuations, ranging from ~−100 to +100 kJ day^−1^ outside episodes of migratory flight (Fig. 3C). While the grand mean of energy balance primarily reflected the allometrically derived estimate of field metabolic rate (fig. S7), peaks during moonlit nights often approached the upper limits set by maximum gizzard capacity and night length, given the constraining role of the nightjars’ limited food processing rate. In contrast, energy balances during new moon periods were generally lower than expected. This reduction was mainly due to decreased foraging activity at dawn, resulting in only partially filled “morning” gizzards (fig. S7). Such reductions may reflect limited food availability or accessibility for nightjars at the end of the night because of the reduced activity of flying insects (*15*), although the precise mechanisms remain to be determined.

**Fig. 4.**
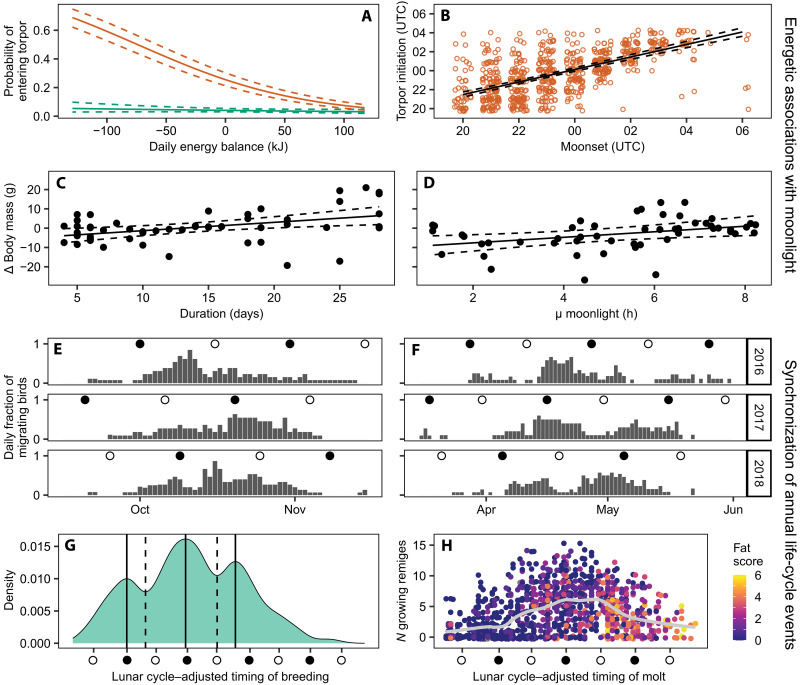
Lunar influences on energetics and annual cycle events of nightjars. (**A**) Probability of individuals entering torpor as a function of daily energy balance during breeding (green) and nonbreeding (orange) seasons. (**B**) Linear relationship between daily moonset time and torpor initiation in the nonbreeding season. (**C** and **D**) Body mass changes in repeatedly captured nightjars fueling for autumn migration in Doñana, relative to the interval between measurements (C) and mean moonlight hours of those nights (D). (**E** and **F**) Daily fraction of tracked birds undertaking nocturnal migratory flights ≥1 hour. Labels indicate the year of autumn migration (e.g., “2016” refers to the 2016–2017 season). (**G**) Temporal distribution of incubating females captured during 2011 to 2020. Near-monthly peaks and nadirs in captures are indicated by solid and dashed lines. (**H**) Temporal distribution of molt intensity, measured as the number of simultaneously growing primaries and rectrices (max. 30) in adults captured during 2011 to 2020. Gray line: smoother LOESS (locally estimated scatterplot smoothing). Point colors denote visually estimated fat stores, ranging from “0” (typical for breeding birds) to “6” (maximal score before migration departure). Open and filled dots [(E) to (H)] on the *x* axis denote full and new moons. To account for interannual variation in lunar timing across the 10 sampling seasons [(E) and (F)], records in (G) and (H) were recalculated relative to the lunar cycle using the first full moon in May as a reference.

To cope with transient energy deficits, such as those occurring during new-moon periods, some caprimulgids have evolved the ability to facultatively reduce their body temperature and enter torpor, thereby minimizing energy expenditure (*7*, *8*, *20*). Using skin temperature data (Fig. 1, C to E, and figs. S2 and S3), we investigated energy-conservation responses in red-necked nightjars. We found that both the timing and magnitude of torpor bouts closely mirrored the annual dynamics of the nightjars’ energy balance (Fig. 3, D and E). This pattern arose from the strong effect of a negative energy balance during the nonbreeding season on both the frequency and intensity of torpor (GLMM: *P* < 0.001; Fig. 4A, fig. S8, and tables S5 and S6) and from a synchronized use of torpor around new moons (GLMM: *P* < 0.001; table S7). Trends in skin temperature further indicated that nightjars on average initiated torpor ~1 hour after moonset (LMM: *P* < 0.001; Fig. 4B and table S8), emphasizing how the loss of moonlight presages declining foraging success and triggers a hypothermic response. Nevertheless, because skin temperature only occasionally dropped below 30°C and torpid birds typically became active at dawn, the nightjars appear to undergo moderate hypothermia confined to the night, thereby achieving modest yet meaningful energy savings (*7*, *8*, *20*).

The apparent absence of a lunar signal in the use of torpor during the breeding season suggests either that nightjars remain above a critical energy intake threshold sufficient to maintain a net energy balance, as indicated by the derived energy balance values in Fig. 3C, or that they sustain constant body temperature at the expense of running periodic energy deficits (*31*). Such cumulative energy gains and losses should produce cyclic body mass fluctuations as energy reserves are depleted and replenished during dark and moonlit nights. Using gizzard-corrected body mass data collected throughout the breeding season, we found that the population mean body mass fluctuated with the lunar cycle, peaking 6 days after the full moon (LMM: *P* < 0.001; table S9). If nightjars rely on lipid stores with an estimated energy content of 39.3 kJ/g (*32*), the mean energy buffer available for new-moon periods is ~207 kJ, or 14 kJ day^−1^, equivalent to 11% of field metabolic rate. This value is likely to vary throughout the breeding season as the energy demands of reproduction decrease and nighttime duration increases (*30*, *31*). Consequently, nightjars should face an increasing lunar cycle effect on energy intake as autumn approaches, manifested as increasing amplitudes of energy balance fluctuations (compare Fig. 3C).

To examine these effects on birds before autumn migration departure (fig. S9), we estimated fuel deposition rates under different moonlight conditions on the basis of repeated body mass measurements of individuals. Fuel deposition estimates accounting for lunar effects indicated that nightjars gained 0.43 ± 0.14 g/day (LMM: *P <* 0.003, *n* = 52 individuals; Fig. 4C and table S10), representing a modest 18% of the allometrically derived maximum fuel deposition rate for birds (*33*). Moonlight had a positive effect on fueling; over an average 14-day monitoring period, nightjars gained an additional 1.44 ± 0.55 g per hour of moonlight (LMM: *P* < 0.009; Fig. 4D). This corresponds to ~0.1 g/day or ~30% of the maximum potential energy intake rate predicted from our measurements of food processing capacity. Given that the duration of darkness (astronomical night) during the fueling period in Doñana is not much longer than the retention time of a full gizzard, the modest effect of the full moon on fueling rates is to be expected, assuming that the gizzard’s storage capacity represents an adaptation that enables crepuscular binge eating and therefore buffers the influence of lunar periodicity.

### Lunar regulation of annual life-history events

Despite adopting multiple strategies to buffer lunar-driven fluctuations in foraging opportunities (e.g., enlarged gizzard capacity, cyclic activity patterns, and body temperature regulation), the persistent lunar signal in nightjars’ energy balance and fueling rates suggests that they trade periodic energy deficits against the fitness costs of these countermeasures, such as the mass-dependent costs of carrying excess digestive loads. Lunar modulation of energy balance should ultimately orchestrate the organization of energy-demanding life-history events, including migration (*16*, *34*), reproduction (*35*–*37*), and feather molt (*38*). Seasonal migrations exhibited clear monthly fluctuations, meaning that most nightjars tracked the lunar cycle to migrate synchronously (Fig. 4, E and F). The timing of spring migration peaked on average 13 days after the full moon (GLMM: *P* < 0.001; table S11). This pattern is consistent with the timing of peak migration in the closely related European nightjar (*Caprimulgus europaeus*), occurring 11 days after the full moon (*16*). In contrast, autumn migration peaked about 1 week before the full moon (GLMM: *P* < 0.001; table S12). This was partly due to autumn departure occurring 5 days later relative to the preceding full moon compared to spring departure (GLMM: *P* < 0.001; fig. S10 and table S13), suggesting season-specific trade-offs in energy allocation resulting from overlapping breeding, molt, and migration. Consequently, autumn migratory flights were less strongly coupled to the lunar cycle than spring flights and occurred to a greater extent during moonlit nights (fig. S10). Despite these seasonal asymmetries, migrating nightjars still tracked the lunar cycle and migrated synchronously, although the factors underlying the seasonal differences remain to be determined.

The capture rate of incubating females primarily reflected the breeding season of nightjars in Doñana (*39*). Nonetheless, we detected peaks and nadirs in numbers associated with the lunar cycle (Fig. 4G and table S14). On average, the probability of capturing a breeding female peaked 10 days after the full moon (GLMM: *P* = 0.02; table S15), a finding consistent with observations from other caprimulgids that adjust reproduction to the lunar cycle (*35*–*37*). Considering an incubation period of 16 to 19 days (*39*), the inferred distribution of egg-laying dates suggests a population-level match between the timing of hatchling emergence and the full moon. Such lunar synchronization of reproduction may optimize foraging conditions and secure the necessary energy intake during this critical stage, thereby increasing chick survival rates and, in turn, the reproductive success of the parents (*35*–*37*). By contrast, we found no significant effect of the lunar cycle on the timing or intensity of flight feather molt (Fig. 4H). We speculate that decisions on the timing and extent of feather shedding, quantified in this study, are primarily governed by a combination of endogenous and exogenous factors other than moon-induced energetic states, such as migratory program and breeding status or success (*2*, *39*).

The effect sizes of the lunar cycle’s influence on the nightjars’ annual life cycles are admittedly small, but we argue that this largely reflects three ecological factors. First, we have shown that the gizzard’s storage capacity effectively buffers the consequences of moon-induced fluctuations in the daily time available for foraging, particularly during the short summer nights (see Fig. 3C and fig. S7). Second, the life-history events of interest occur over discrete and relatively brief periods and may therefore only occasionally be affected by the lunar cycle (*15*). For instance, an individual nightjar completes its full migratory route in about a week, yet population-level analyses of migration encompass multiple lunar cycles; the same applies to females carrying a detectable egg (compare Figs. 1B and 4, E and F). Third, the temporal overlap among annual cycle events likely obscures potential lunar signals at the population level, particularly in the breeding season, as any surplus energy may be simultaneously allocated to multiple competing demands (*38*, *39*). In contrast to the nonbreeding season, GPS-tracked nightjars remained mobile during dark nights of the breeding season (LMM: *P* = 0.001; table S16), a pattern that we interpret as complementary off-site foraging activity by breeding individuals, as also reflected in our gizzard and activity data (Figs. 2C and 3, B and C, and fig. S11). The role of artificial light sources in the surrounding landscape in facilitating nightjar foraging on dark nights remains unclear in our study system, although such effects have been documented in closely related species (*40*, *41*). This question is currently the focus of ongoing research.

## DISCUSSION

By combining a unique long-term monitoring dataset with individual-based data collection using state-of-the-art miniaturized MDLs, we unraveled intricate relationships between the internal energetic state of nightjars and key annual cycle events governed by the lunar cycle. The energetic state of individuals may act as an internal signal enabling nightjars and other organisms to integrate short-term fluctuations in ecological conditions (e.g., moonlight, microclimate, and food resources) into fitness-related decisions. For instance, reaching specific energetic thresholds may signal favorable phases of the annual cycle (e.g., the accumulation of reserves required for egg formation) and trigger reproductive decisions at opportune times (e.g., clutch initiation during periods of profitable foraging), even in the absence of external cues or endogenous clocks. Further research, including lunar free-running experiments, as well as simulation- and field-based approaches, is required to determine the importance of energetic feedback and other time-keeping mechanisms in nocturnal species exposed to the superimposition of diel, seasonal, and lunar periodicities.

Our findings provide support for the occurrence and interplay of multiple adaptations to lunar periodicity in nightjars, enabling them to endure periods of famine around the new moon. These adaptations include the ability to enter hypothermia, to cyclically replenish body reserves, and to maintain a large food-storage capacity for binge feeding during brief crepuscular periods. We show that such lunar-related countermeasures are used only partially or temporarily, presumably due to fitness costs that vary across the annual cycle (*20*), resulting in predictable lunar synchronization of life-history events.

Nightjars occupy a pivotal trophic position as both predators of insects and prey for larger animals. Consequently, lunar-driven fluctuations in their daily foraging activity and energy balance may cascade through the community, indirectly affecting prey populations and predator-prey interactions and, ultimately, broader ecosystem functioning (*9*, *42*). Our study underscores the importance of moonlight for life in the dark and reveals potential adaptations that enable organisms to exploit the nocturnal niche. It also stresses the need to integrate ecological energetics into annual-cycle tracking studies of nocturnal organisms as an essential step to advance nighttime ecology, particularly as an increasing number of species across the globe become nocturnal in response to anthropogenic disturbance and climate change (*43*, *44*).

## MATERIALS AND METHODS

### Data collection

This study combines field measurements collected during 2011 to 2020 as part of a long-term study of red-necked nightjars (henceforth, nightjars) in the Doñana National Park (37°7′N, 6°33′W) and biologging data from MDLs and GPS tags used for full annual cycle tracking between 2016 and 2020. Only adults are considered in this study because they constitute most annual captures, and their recapture rates from 1 year to the next (~45%) are approximately twice those of juveniles (~25%). Details on the study site and trapping methods can be found in (*45*). For each bird, we recorded body mass (to the nearest 0.1 g), the amount of subcutaneous fat (0 to 6 scale), the size of the gizzard (0 to 4 scale; see figs. S4 and S5), the total number of primaries and rectrices in active molt, and the stage of brood patch development in males and females (both sexes incubate in this species) using a 0-to-5 scale [0, no brood patch; 1, defeathering started; 2, defeathered patch but no engorgement of the skin; 3, presence of an egg in the abdomen (females) and/or full brood patch of engorged (males and females) and vascularized (females) skin; 4, regressing skin engorgement; 5, patch refeathering].

### Bird tagging

Between 2016 and 2018, a total of 74 custom-made MDLs were used to track 56 different adults (34 females and 22 males) throughout one to three annual cycles. These tags monitor the flight activity of individuals by sampling wingbeat-induced variations in vertical acceleration (*16*). In addition, 10 adults (5 females and 5 males) were equipped with GPS loggers (nanoFix-GEO-mini, 2 g, Pathtrack Ltd.) in 2018. The tags were mounted on the bird’s back using a full-body harness consisting of four nylon straps that could be joined and adjusted independently over the sternum before being stitched together permanently. MDLs and GPS loggers (2 to 2.2 g including the harness) represented <3% of the total body mass of adult nightjars. Between 2017 and 2020, we retrieved a total of 40 MDLs (54%) and 5 GPS tags (50%). No signs of feather or skin abrasion resulting from the harness were detected after careful inspection of recaptured individuals. The analyses performed in this study are based on data from 34 MDLs and 5 GPS loggers (30 different individuals) after exclusion of data from 6 MDLs because of partial or total technical failure. We followed all applicable international, national, and institutional guidelines for the capture and marking of animals. The study was approved by the relevant regional authorities, most recently under license no. REGA 410910008014. Capture and bird-tagging procedures conducted during the long-term study were approved by the CSIC Ethics Committee (protocol no. OH 1098/2021) and complied with Spanish and European legislation on the protection of animals used for scientific purposes.

### GPS and MDL data acquisition and processing

We used GPS data to determine the annual space use and migration phenology in a subset of individuals and to describe general characteristics of their movement ecology. To distinguish stationary periods from migratory flights, we calculated daily translocation distances as the great-circle distance between consecutive GPS fixes (*46*). We then classified periods as stationary or in movement using a 10-km threshold, given that caprimulgids can commute several kilometers while foraging during stationary periods (*45*, *47*, *48*). MDL tags were programmed to sample flight activity through acceleration in the *z* axis. Each sample was recorded over 100 ms at 100 Hz in the range of ±4 g. The mean value was subtracted from each of the 10 measurements to account for static gravity, and activity was classified as flight if at least three of the 10 records exceeded |*g*/3|. For MDLs deployed in 2016 and 2017, sampling was repeated 10 times at 5-s intervals, with the sampling procedure running every 5 min. For those deployed in 2018, sampling was repeated five times at 5-s intervals (see Supplementary Text).

Flight activity data were used to distinguish periods of migratory flight from those of presumed foraging and inactivity. Migratory flights are usually readily visible in nightjar actograms as extended periods of continuous, elevated activity lasting several hours [figure 2 in (*11*)]. These periods of presumed migratory flights were extracted from the activity data recorded by the MDLs, as described by Norevik *et al.* (*16*). Hours of generally high activity (values ≥6 for 2016 and 2017 tags or ≥3 for 2018 tags) and no zero records were classified as core flight periods. The number of 5-min intervals with activity values ≥6 (2016–2017 tags) or ≥3 (2018) recorded during the hour immediately before and after each core period was added to the ends of the core segment to define the total duration of migratory flight.

Following Norevik *et al.* (*16*), all activity recorded outside presumed migratory flight segments was defined as potential foraging activity. This approach enabled quantification of the maximum potential hourly foraging activity of nightjars. The hourly foraging effort was expressed as the proportion of positive activity recordings relative to the total number of samples (*n* = 120 or 60 depending on the tag version). To assess patterns of foraging activity during the breeding season, we extracted MDL data from spring arrival to autumn departure. Sunset times and lunar illumination at the study site were obtained using the R package “suncalc” (*49*). Data were restricted to 1 hour before sunset to 2 hours before sunrise and classified as moonless or moonlit on the basis of the fraction of illuminated moon (new moon: 0 to 15%; full moon: 85 to 100%; see Supplementary Text).

### Measurements of body temperature

MDLs included a temperature sensor placed on the bird’s skin on the back that recorded skin temperature data on a 1-hour basis. Because dorsally mounted tags are exposed to solar heating during the day, analysis of skin temperature data from temperature sensors was restricted to nighttime. We excluded periods of migratory flight on the basis of activity data (see above), as records may be influenced by ambient temperature declines associated with altitude changes (Fig. 1 and fig. S2). Last, we separated skin temperature data between hours with and without activity, the latter potentially reflecting periods of heterothermy or torpor. From the temperature sensor data, we derived summary statistics of skin temperature during active nighttime hours for subsequent analyses of lunar cycle–associated variation and for comparison with published skin temperature data from other caprimulgids (table S1).

### Calculation of maximum gizzard capacity and food processing rates

Caprimulgids lack a true crop but use the gizzard as a food storage organ (*19*, *24*). Therefore, the maximum potential capacity of a full gizzard provides a reliable indication of the physical limits to food consumption in nightjars (*24*). Estimating the gizzard capacity also enabled us to quantify the consumption component of the energy balance of nightjars after accounting for processing rates (see below). The maximum potential gizzard capacity (in grams) was estimated from body mass data as the difference between the average mass of individuals with an empty gizzard (score 0) and the average mass of individuals with a full gizzard (score 4). Only birds with a fat score of zero and lacking a brood patch or an egg were considered to avoid the confounding effect of fluctuations in body mass because of fuel stores and breeding. Usually, the amount of fat is estimated visually according to some classification scale (0 to 6 in our case), but the relationship between fat classes and the actual amount of fat may not be linear, and also, the transitions between some fat classes may be blurred (*50*, *51*). This uncertainty also applies to the classification of brood patch development. Hence, restricting the sample to lean, nonbreeding birds ensures more precise estimates of digestive capacity. The maximum potential gizzard capacity in this study is estimated from average cross-sectional data collected across different times of the season and different years, minimizing the confounding influence of potential plasticity in digestive capacity (*6*).

Food intake is influenced by the instantaneous rate of food acquisition (i.e., storage capacity) and also by the rate of food processing. Estimating the food processing rate is therefore required to assess the physiological limits to food consumption and thus estimate the daily energy intake. To estimate the food processing rate under field conditions, we used repeated captures of free-ranging individuals to measure the rate of body mass loss after food consumption, as described in (*29*). Our estimate of food processing rate is based on 19 adult individuals captured and measured for body mass (and also for gizzard in 15 of them) more than once during the same night, taking advantage of the unintentional postcapture cessation of foraging (*45*). Only measurements taken at time intervals ≥20 min were considered to make sure that a measurable amount of material had been excreted in the feces (*29*). None of the individuals measured for gizzard size had zero gizzard scores at first measurement, confirming prior food consumption (mean gizzard score, 2.6; range: 1 to 4); also, their gizzards still contained some food at the second measurement (mean gizzard score, 1.4; range: 1 or 2), minimizing the possibility of underestimation of mass loss rate (*29*).

The food processing rate reflects the amounts of both absorbed and unabsorbed materials. The absorption rate in other invertebrate-eating birds has been determined to be around 11% (*29*). Mass loss from the first to second capture is assumed to be primarily caused by the evacuation of unabsorbed material (defecation, *D*), given that evapotranspiration during the night is probably negligible (*29*). Food processing rates for individual nightjars were estimated from the difference in body mass from the first to second measurement divided by the time between captures, which is defecation rate (DR), assuming an absorption fraction (*f*_a_) of 0.113 (*29*), thus

$$\text{Food processing rate}=\text{DR}\times {\left(1-f_{a}\right)}^{-1}$$

To measure the maximum potential food processing rate in the study population, we focused on the individuals representing the upper quartile of the food processing rate distribution (*n* = 5) after excluding ID 1B45863, because the estimated rate in this particular bird almost doubled that of the bird with the next highest food processing rate and is therefore considered a likely measurement error (table S4).

### Calculation of daily energy balance

To investigate the relationship between the lunar cycle and nightjar behavior, we parameterized individual-based models to calculate daily energy gain or loss. Briefly, we calculated a simplified daily energy balance on the basis of daily energy gains from foraging and costs associated with flight activity and allometrically derived field metabolic rates (see below). The model outputs are not intended to quantify actual daily energy balances but to provide realistic baseline estimates across the annual cycle from which systematic deviations (e.g., lunar cycle–related fluctuations) may occur. This approach allows identification of periods of high energy expenditure resulting from continuous flight (e.g., migration) and periods of energy deficit caused by poor foraging conditions (e.g., around the new moon). Because nightjars are mainly nocturnal, the daily energy balance was calculated from midday to midday, rather than midnight to midnight. Hourly energy intake (*Ei*_(t)_) was calculated on the basis of the energy content of a noctuid moth [*E*_m_ = 27.2 kJ × g^−1^ dry mass (*52*)], the average prey of nightjars (*18*), and a dry-to-wet ratio (*R*_dw_) of 0.36/1 (fig. S6). The dry mass of nightjar prey was estimated from the fresh gizzard contents of five road-killed individuals obtained from regional recovery centers. Gizzard contents were weighed (± 0.01 g) immediately after extraction and dried to constant mass (7 to 9 days at 60°C). On average, gizzard contents consisted of 64.1% ± 3.5 (SE) water (fig. S6), consistent with the 64.2% water content previously reported for individual noctuid moths (*52*).

*Ei*_(t)_ was adjusted to hourly metabolizable energy intake (*MEi*_(t)_) to account for an average metabolized energy coefficient of 0.77 for arthropods (*53*). Using the above approach allowed us to account for both external and internal constraints related to the nightjar’s energy intake (*54*). No extra costs in feeding efficiency due to the fullness of the gizzard were considered, given that nightjars are assumed to use their maximum storage capacity only when anticipating a sharp reduction in food intake. *MEi*_(t)_ depends on the mass of gizzard content (*Mgi*), which varies between 0 (empty) and a predefined maximum (*Mgi*_max_; see the previous section). The gizzard content is regulated by the hourly food intake (*F*_i(t)_) and food processing (*F*_p(t)_), respectively

$${\mathrm{Mgi}}_{\left(t\right)}={\mathrm{Mgi}}_{\left(t-1\right)}+F_{i\left(t\right)}-F_{p\left(t\right)},0\geq {\mathrm{Mgi}}_{\left(t\right)}\leq {\mathrm{Mgi}}_{\text{max}}$$

The hourly food intake is calculated as

$$F_{i\left(t\right)}=\text{FR}\times t_{f\left(t\right)}$$

where FR is the foraging rate based on the time (*t*_max_ = 10 min) required to fill the gizzard, i.e., <15 to 20 min [see (*19*) and references therein]. Thus

$$\text{FR}={\mathrm{Mgi}}_{\text{max}}\times {t_{\text{max}}}^{-1}$$

The time per hour spent foraging (*t*_f(t)_) is based on the fraction of activity samples that registered a presumed foraging activity signal. The hourly throughput (*F*_p(t)_) of a nightjar gizzard is based on the gizzard content available for retention (*Mgi*_(t)_) and is ultimately limited by the food processing rate (see the previous section).

Energy expenditure per hour as referred to in the model is the combined costs of field metabolic rate and flight activity recorded by the MDL. We have not found any empirical data on field metabolic rate for the red-necked nightjar or related species but calculated realistic field metabolic rate estimates from an allometric regression for birds living in arid environments (*55*)

$$\begin{array}{c} \text{Field metabolic rate}\;\left(\text{kJ}\;{\text{day}}^{-1}\right)=\\ 6.35\times {\mathrm{Mb}}^{0.671},P<0.0001,R^{2}=0.957 \end{array}$$

where *Mb* is the body mass (g). Using a body mass of 87.13 g, which is the average lean mass of nightjars in the study population (*n* = 242 males and 327 females), resulted in a field metabolic rate of 127.24 kJ day^−1^. To validate the calculated field metabolic rate, we used an allometric regression to calculate the basal metabolic rate as follows (*56*)

$$\begin{array}{c} \text{Basal metabolic rate}\;(\text{ml}\;O_{2}\;g^{-1}\;{\text{hour}}^{-1})=\\ 6.13\times {\mathrm{Mb}}^{-0.422},F_{1,9}=6.95,P<0.05,R^{2}=0.46 \end{array}$$

The oxygen consumption rate was converted into kJ day^−1^ using the following formula

$$\begin{array}{c} \text{Basal metabolic rate}\;(\text{kJ}\;{\text{day}}^{-1})=\\ \text{basal metabolic rate}\left(\text{ml}\;O_{2}\;g^{-1}\;{\text{hour}}^{-1}\right)\times \mathrm{Mb}\times 24\times 10^{-3}\times c \end{array}$$

where *c* is an oxygen equivalent of 20.51 kJ/liter O_2_ (*57*). The derived value for a basal metabolic rate of 39.91 kJ day^−1^ corresponds to 31% of our field metabolic rate estimate, consistent with field-to-basal metabolic rate relationships observed in birds and mammals (*58*).

Energy expenditure in locomotion was derived by using the R package afpt (*59*), and the average wing measurements are provided in table S17. The afpt model, based on the actuator disc model (*60*), incorporates a more realistic calculation of induced power by accounting for the wake topology produced by flapping wings (*59*). Given the U-shaped relationship between energy output and flight speed, migrating birds are expected to fly at speeds that minimize energy cost per unit distance. For the red-necked nightjar, a radar-recorded flight speed of 10 m s^−1^ (*61*) closely matches predicted migratory flight speeds (*U*_mr_; table S17). Outside migration, nightjars use flight for commuting between diurnal roosts and nocturnal foraging sites, pursuing prey, and escaping predators (*45*, *62*). These nonmigratory flights are presumed to be more energy demanding per unit time than cruising flight because of frequent take-offs, landings, and acceleration during prey pursuit. Therefore, we increased the energy costs of presumed foraging flights (*E*_f_) by 25% relative to that of migration flight (*E*_m_), yielding an hourly energy expenditure

$${\mathrm{Ee}}_{\left(t\right)}={\text{FMR}}_{h}+E_{\text{mr}}\times t_{\text{mr}\left(t\right)}+E_{f}\times t_{f\left(t\right)}$$

where *t*_mr_ is the estimated amount of time per hour spent migrating, and *t*_f_ is the hourly fraction devoted to foraging. Thus, the hourly energy balance HEB_(t)_ is calculated as

$${\text{HEB}}_{\left(t\right)}={\mathrm{MEi}}_{\left(t\right)}-{\mathrm{Ee}}_{\left(t\right)}$$

### Deriving lunar cycle–associated temperature data

We quantified the nighttime variation in skin temperature as recorded by the MDLs using two approaches: First, we calculated a heterothermy index (HI)

$$\text{HI}=\sqrt{\frac{\Sigma {\left(T_{\text{sk}-\text{mod}}-T_{\text{sk}-i}\right)}^{2}}{n-1}}$$

where *T*_sk-mod_ is the modal skin temperature, *T*_sk-*i*_ is the skin temperature recorded at time *i*, and *n* is the daily sample size of skin temperature per MDL (*63*). The HI provides a simple metric to quantify variation in skin temperature relative to the typical individual skin temperature recorded by the MDL (*T*_sk-mod_) and has previously been used to successfully capture lunar cycle–related variation in skin temperature in nightjars (*8*). We also identified instances of torpor, an energy-saving state characterized by reduced metabolic rate and a controlled reduction in body temperature during inactivity (*7*). Periods without activity registrations were scanned for reductions in skin temperature. To account for individual differences in skin temperature measurements due to, e.g., MDL deployment variation, torpor onset was defined as values 2× SD below the mean (table S1), corresponding to ~35°C. Because this may represent a conservative threshold, analyses were repeated using 3× SD below the mean (~33°C), yielding consistent results.

### Calculation of fuel deposition rates

To calculate the fuel deposition rate, we first estimated the onset of autumn fuel deposition using a breakpoint analysis of seasonal fat score trends (fig. S9) (*64*). From this subset, we selected individuals measured ≥2 times. The fuel deposition rate was then calculated as the change in body mass between the first and last measurements, divided by the number of days between measurements (*65*, *66*). Before analysis, body mass measurements were corrected for food ingested using gizzard scores and gizzard mass estimates, assuming a linear relationship between the gizzard score and the content mass. Breeding males and females (brood patch score, 3) were excluded to avoid the confounding effects of additional mass from the egg or brood patch.

### Deriving life-cycle data

To analyze lunar effects on migration intensity, we followed the procedure described by Norevik *et al.* (*16*). We defined migratory flight nights as those when MDLs ≥1 hour of continuous flight or when GPS data indicated movement >10 km. For each date, we calculated the fraction of tagged individuals that migrated. To assess lunar synchronization of reproduction, we focused on the incubation period inferred from brood patch data of females. Because exact laying dates could not be precisely determined because of the difficulty of finding nests, we used the number of incubating or gravid females (brood patch score, 3) captured during systematic surveys to describe temporal patterns of reproduction at the population level (*39*). To evaluate lunar effects on molt intensity, we used data on the number of simultaneously growing primaries and rectrices in captured birds. Molt primarily occurs at the end of the breeding season, and birds may allocate energy to reproduction and feather replacement simultaneously (*39*). Molt analysis was restricted to dates from 29 July onward, covering the main molt period identified by a breakpoint analysis of the seasonal trend in molt intensity (*64*).

### Statistical analysis

LMMs and GLMMs were fitted using the R package “glmmTMB” (*67*) in R version 4.2.2 (http://cran.r-project.org). To compute *P* values for individual predictors, we compared full models with reduced models, excluding each predictor, using likelihood ratio chi-square tests. Model assumptions were checked using the R package DHARMa (*68*), and model fit was assessed using conditional *R*^2^, which represents the proportion of variance explained by both fixed and random effects (*69*). Conditional *R*^2^ values were computed using the r.squaredGLMM function in the MuMIn package (*70*). For analyses of lunar cycle effects, we used data on the fraction of illuminated moon surface (0, new moon; 1, full moon), resulting in a near-sinusoidal signal corresponding to the date associated with the dependent variable. To explore potential phase shifts in moon-associated patterns, we reran models with time-shifted moon-phase data until the phase yielding the highest conditional *R*^2^ value was identified.

#### Daily activity

To model the probability of nocturnal activity in relation to the moon phase, we used a GLMM (binomial distribution of errors and log-link function). To assess the effect of moon phase on the activity of the birds, we constructed a model containing the time relative to sunset as a continuous covariate and the factorial variable “Moon phase” and their interaction as main predictors. We included bird identity as a random intercept to account for repeated measurements of the same individuals and time relative to sunset per bird identity as a random slope.

#### Gizzard fullness

To analyze nighttime trends in the degree of gizzard fullness in relation to the moon phase, we used an LMM with gizzard score (0 to 4) as the response variable, time relative to sunset as a continuous covariate, and the factorial variable “Moon” and their interaction as the main predictors. We included bird identity and date as random intercept effects to account for the effects of repeated observations of the same individuals (*n* = 953 captures of 634 individuals) and unmeasured environmental factors (e.g., temperature and food availability) on the day of capture (*n* = 186 days). No gizzard data are available for the first 1 to 2 hours after sunset and before sunrise because of restrictions from the night-lighting technique used for capturing nightjars. For the same reason, the trends in gizzard fullness around the morning peak of activity could not be examined.

#### Body temperature

To analyze the effect of daily energy balance on HI in breeding and nonbreeding season, we used an LMM with HI (log_10_-transformed, continuous variable) as a response, daily energy balance as a continuous covariate, and the factorial variable “season” and their interaction as main predictors. We included bird identity as a random intercept to account for repeated measurements of the same individuals and daily energy balance per bird identity as a random slope. To model the probability of torpor in relation to daily energy balance, we used a GLMM (binomial distribution of errors and log-link function). To assess the effect of daily energy balance on the probability for the birds to enter torpor, we constructed a model containing daily energy balance as a continuous covariate and the factorial variable “Season” and their interaction as the main predictors. We included bird identity as a random intercept to account for repeated measurements of the same individuals and daily energy balance per bird identity as a random slope.

#### Body mass variation and fuel deposition rates

To analyze the effect of lunar cycle on body mass variation, we used an LMM with body mass corrected for gizzard content as a dependent continuous variable and lunar cycle as a continuous predictor. We included bird identity and year as random intercepts to account for repeated measurements of the same individuals and for systematic differences between breeding seasons. To analyze the effect of moon phase on the rate of mass gain of nightjars, we used an LMM with difference in mass corrected for gizzard content as the response variable and the duration between measurements and average nighttime duration of moon illuminated throughout the fueling period as main predictors. We included “year” as a random intercept to account for systematic differences between breeding seasons.

#### Migration, reproduction, and molt

The probability of initiating a migratory flight and the probability of capturing an incubating female in relation to the lunar cycle were both modeled using a GLMM with a binomial error distribution and log-link function, including the lunar cycle as a continuous covariate. Bird identity and year were included as random intercepts to account for repeated measurements of the same individuals and for systematic differences among breeding seasons. To evaluate the degree of modality in the occurrence of incubating females relative to the lunar cycle, we first transformed trapping dates into lunar cycle days using the date of the first full moon in May of each year as a reference. We then used the modetest and locmodes functions in the multimode R package (*71*) to test for multimodality and to determine the timing of modes and antimodes in the occurrence of incubating females relative to the lunar cycle. To model the lunar cycle effect on molt intensity, we used a GLMM with a negative binomial error distribution, including lunar cycle as a continuous covariate and breeding status (incubating/nonincubating) as a categorical predictor. Bird identity and year were again included as random intercepts.

## Supplementary Materials

### This PDF file includes:

Supplementary Text Figs. S1 to S11 Tables S1 to S17 References
