# Supplementary material for: Moonlight drives the energy balance and annual cycle of a nocturnal forager
Source: Sci Adv. 2026 May 1;12(18):eaed8204. doi: 10.1126/sciadv.aed8204 (PMC13134586; doi:10.1126/sciadv.aed8204)
Supplement: Supplementary file 1 — Supplementary Text Figs. S1 to S11 Tables S1 to S17 References [file sciadv.aed8204_sm.pdf]

Science Advances
AAAS

Supplementary Materials for

Moonlight drives the energy balance and annual cycle of a nocturnal forager

Carlos Camacho *et al.*

Corresponding author: Carlos Camacho, ccamacho@ebd.csic.es;
Anders Hedenström, anders.hedenstrom@biol.lu.se

Sci. Adv. **12**, eaed8204 (2026)
DOI: 10.1126/sciadv.aed8204

This PDF file includes:

Supplementary Text
Figs. S1 to S11
Tables S1 to S17
References

Use of moon phase as a measure of lunar influence

To assess lunar influences on nightjars, we focused on the fraction of the moon surface being illuminated, often referred to as simply 'moon phase', rather than using more complex and sophisticated estimates of on-ground moonlight illumination (see *72*). The reasons for this are several: (*i*) this study aims to explore general periodic patterns across seasons related to the lunar cycle, rather than measuring time- and location-specific effects of light levels on nightjars, (*ii*) moon phase is an easier measure to interpret and is commonly used in ecological studies, facilitating comparison of results; (*iii*) moon phase *alone* accurately predicts activity levels in this and other closely related species (*15, 34*; but see *7*), (*iv*) moon brightness is not a linear function of moon phase, yet for ease of interpretation, and to accommodate differences in the temporal resolution of different data sources (from minutes to days), the results are presented as comparisons of opposite lunar phases (0-15% *vs*. 85-100% of the moon surface illuminated), corresponding to ~7-day periods of contrasting brightness conditions (see Fig. 3 in *72*); (*v*) By the same token, factors affecting the amount of moonlight available to nightjars during different parts of the night, such as moon altitude, are unlikely to influence nightjar responses measured at coarser temporal scales.

Natural history and ecological details of the study species

Many aspects of the natural history and ecology of red-necked nightjars have been described in detail in previous studies (*39, 45, 62, 73, 74*). Therefore, only a brief summary of aspects relevant to the present study is presented in this section.

Foraging ecology

Nightjars are central-place foragers, meaning that they are tied to a central place (i.e., the nest location during the incubation and chick-rearing periods) and return to this location after each foraging trip. Nightjars breeding in our study area commute distances of 3-10 km on a daily basis in search for foraging patches in agricultural areas or near marsh edges (*45, 75*). Nightjars are strictly insectivorous birds. Moths, particularly the pine processionary moth (*Thaumetopoea pityocampa*), make up the majority of prey items of nightjars in the study area (*18*, *39*). The main foraging mode of red-necked nightjars implies the pursuit from the ground of individual targets that are detected in silhouette against the background of the sky. Therefore, some source of illumination is required for efficient nocturnal foraging.

Breeding ecology

The red-necked nightjar is a summer visitor to Spain. Most individuals arrive in the study area during May and, although some clutches can be found in early May, the majority of them do not initiate reproduction until June (*39*). Most (80%) nightjars reproduce for the first time at age 1 year (*73*). They produce 1-2 clutches of 1-2 eggs per breeding season, and replacement clutches after nest failure are common. The incubation period is 16-19 days. Females are responsible for incubation during the day, and both sexes share incubation duties during the night. Chicks fledge at 18–22 days of age, but may continue to receive parental care beyond 35 days of age (*39*).

Migration ecology

The location of the non-breeding grounds and timing of migration of red-necked nightjars are documented in this study for the first time. Estimates of fueling rates before the first migratory flight are available for juveniles in the study population. Daily fuel deposition rates of juveniles

prior to autumn departure range from a maximum of 2.8% of their body mass to zero mass gain or even mass loss (*74*).

Molt

Molt strategies in nightjars are complex, but general differences among age classes have been described. Birds in their first calendar year may undergo a post-juvenile partial molt before autumn departure, including body contour feathers and, exceptionally also 1-2 inner secondaries and greater coverts, but not median or lesser coverts. Birds in their second calendar year replace a small number of flight feathers (e.g., the central pair of rectrices, 1-2 outermost secondaries, 1-2 innermost primaries, and the corresponding upper coverts) before returning to their breeding grounds for the first time. First-time breeders therefore display two generations of feathers. Upon completion of breeding in late summer, all nightjars undergo an extensive post-nuptial molt of up to 10–15 flight feathers (*39*) and replace the rest of feathers on the non-breeding grounds (*76*). From that moment on, the molting sequence is the same as that described for first-time breeders.

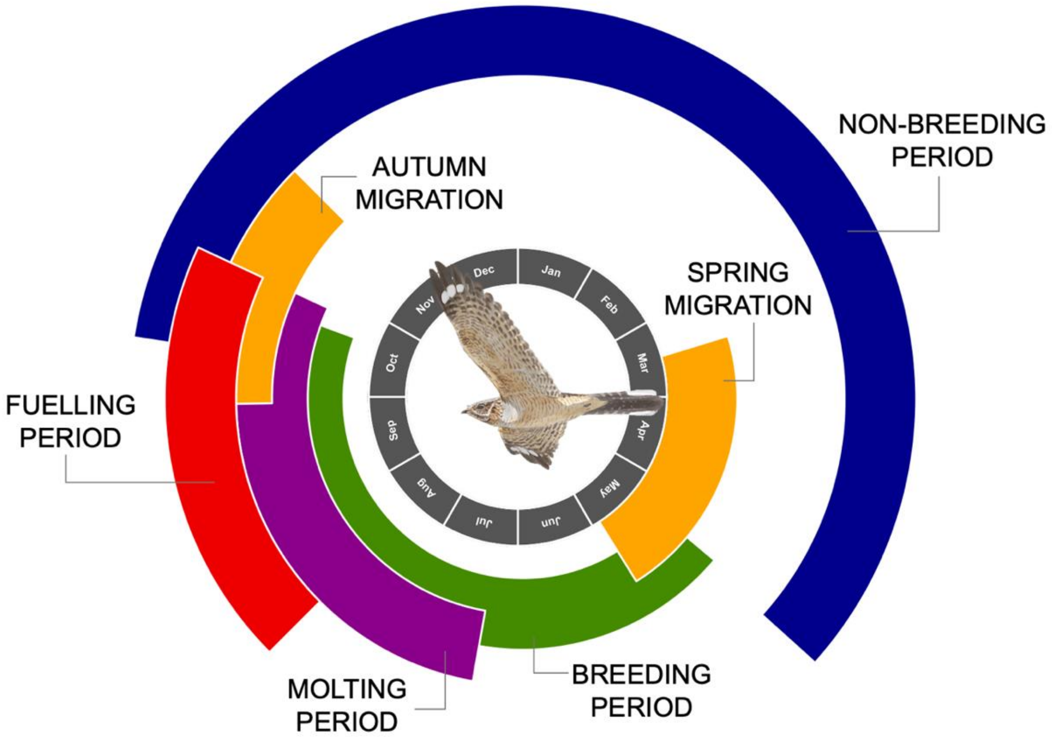

Fig. S1.

Temporal distribution of the main annual cycle stages of the study population of red-necked nightjars, illustrating the strong temporal overlap between stages. The spring migration of the population takes place over a span of 2.5 months, beginning in mid-March (first departure dates from wintering areas) and extending to late May (late arrival dates in Doñana. The breeding season (i.e. incubation, brooding and chick rearing) lasts over 5 months, from the beginning of May (first clutches) to the end of October (independence of the last chicks). The first individuals begin to molt around mid-July and molting birds are recorded until the end of the field season in late October (ca. 3.5 months). Fueling spans from mid-August to the end of the field season (ca. 2.5 months). First departures on autumn migration take place in late September and it takes until mid-November for the last individuals to complete their journey (ca. 1.5 months). Field data for the fueling and molting periods prior to the spring migration are not available, since data collection is limited to the breeding area in Doñana. Nightjar illustration: ©Antonio Ojea, used with permission.

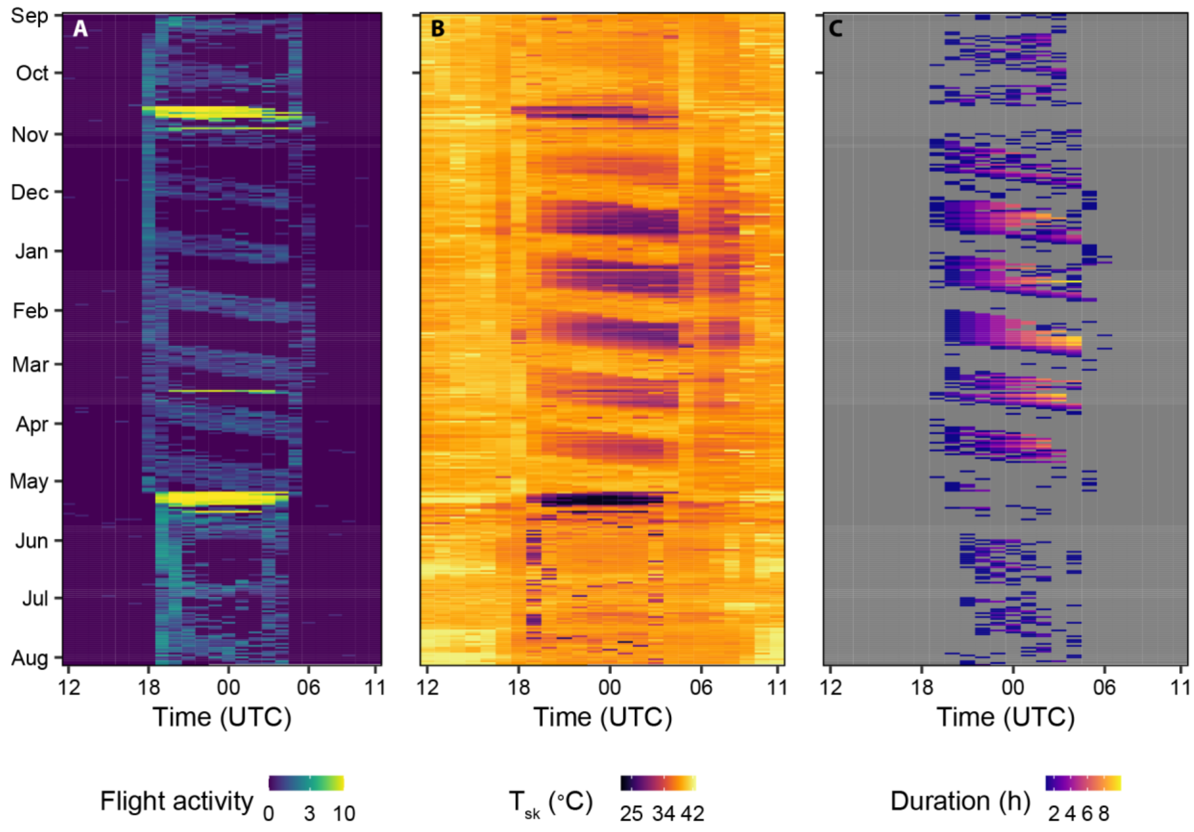

Fig. S2.

Example actogram showing data from logger #XD42. Subplots **(A)** and **(B)** display hourly activity and skin temperature, respectively, as in Fig. 1, B and C of the main text. Subplot **(C)** shows periods of nighttime inactivity, where the color gradient represents the number of consecutive nighttime hours with decreasing skin temperature. All 34 actograms generated in this study are available in the Multisensory data overview file deposited in Mendeley Data, V1, doi: 10.17632/srh8sc547s.1.

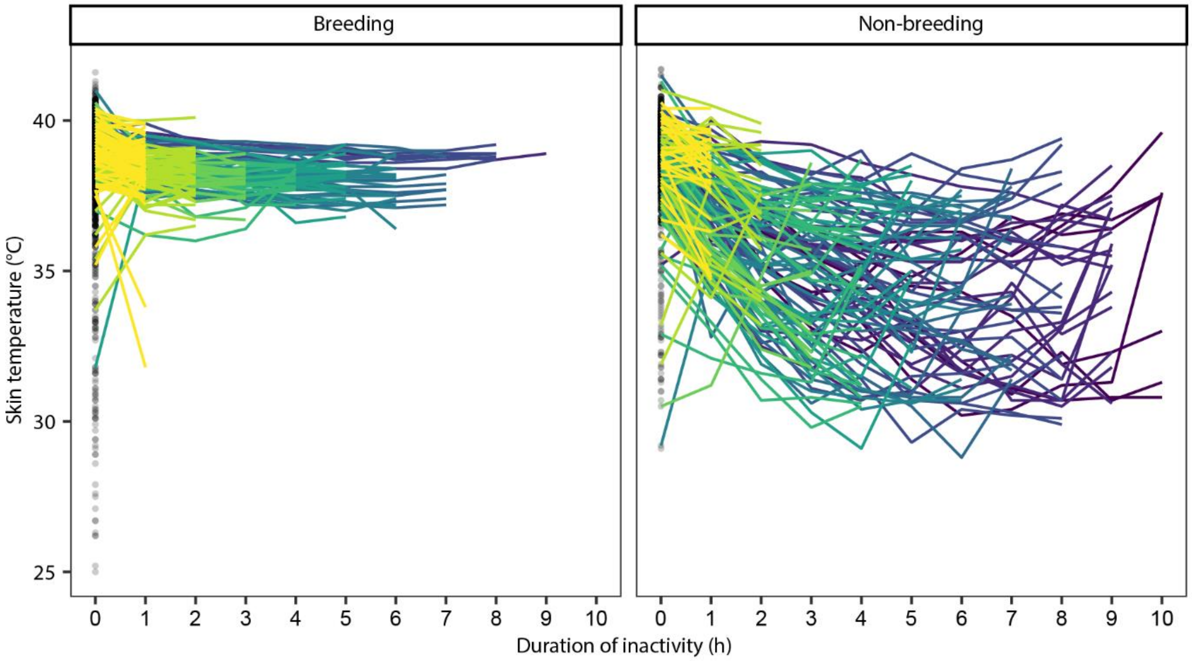

Fig. S3.

Trends in nighttime skin temperatures during periods of inactivity in breeding and non-breeding seasons recorded on a single red-necked nightjar. Data correspond to temperature data presented in Fig.1D-E. Duration '0' represents hours when the birds were active, as recorded by the MDLs. Line colors refer to duration of inactivity to facilitate interpretation. In contrast to the breeding season, inactivity periods during the non-breeding season are often associated with notable temperature drops. For some records, the sensor captured the final increase in skin temperature before the bird became active as registered by the accelerometer.

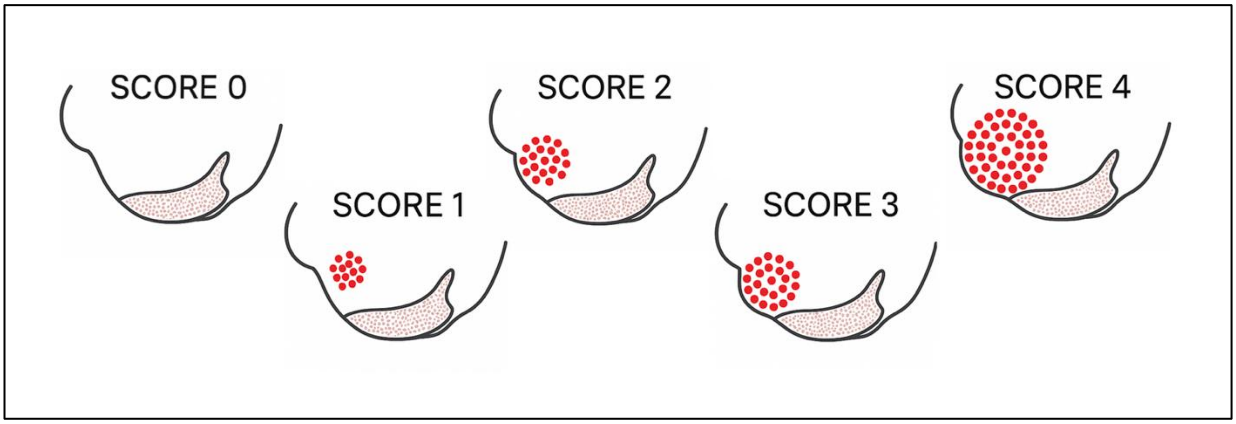

Fig. S4.

Field estimates of the degree of stomach (gizzard) fullness. Mid-sagittal cross-section of the body of a nightjar describing the categories used to score gizzard fullness through palpation of the abdomen, adapted from Jackson (*51*). Based on the degree of softness to the touch, the amount of 'free' space in the abdominal cavity, and the degree of protrusion of the gizzard (red dotted circle) relative to the line of the sternum (shaded elongated area), the degree of gizzard fullness was approximated to a three-dimensional sphere and scored on an ordinal scale as 'score 0' (empty; no lump is felt after applying firm pressure through the abdominal wall), 'score 1' (¼ full, a small, soft lump can be felt by applying medium pressure through the abdominal wall), 'score 2' (½ full, readily tactile lump after applying medium pressure through the abdominal wall), 'score 3' (¾ full; visible protuberance that occupies considerable space in the abdominal cavity, yet the gizzard does not protrude over the line of the sternum), and 'score 4' (completely full; no space in the abdominal cavity and visible protuberance that clearly protrudes over the line of the sternum).

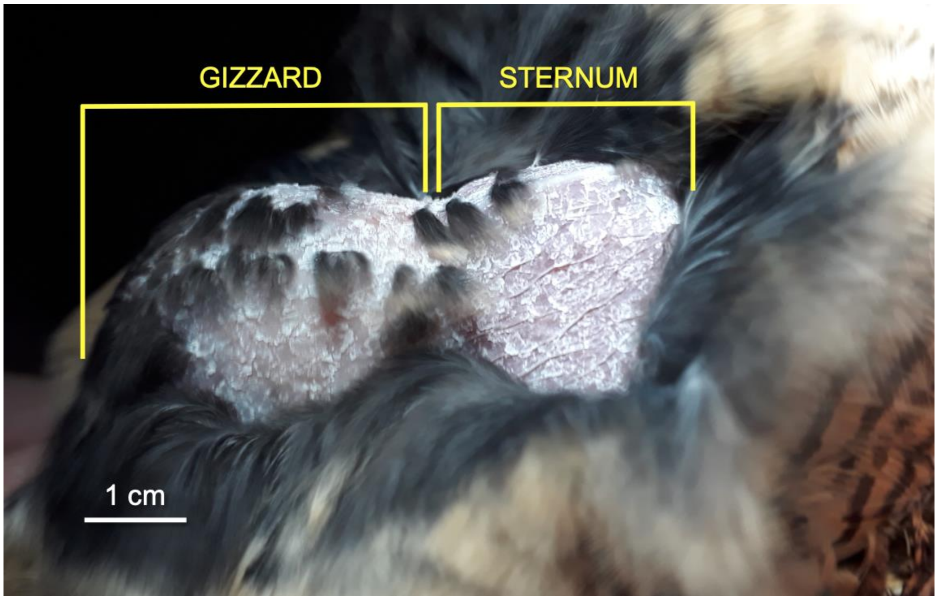

Fig. S5.

Lateral photograph of a full mechanical stomach (gizzard). The full gizzard (score 4) of a nightjar is clearly visible as a large protuberance (approx. 2.5 × 3.5 cm) that protrudes over the line of the sternum. Photo by Carlos Camacho.

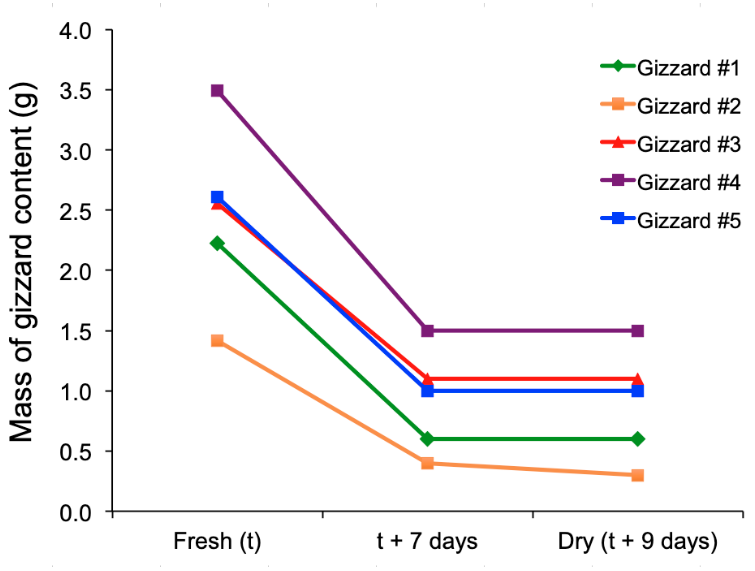

Fig. S6.

Change in mass of the fresh contents of five gizzards after dehydration. Gizzard contents consisted of 57-73% water. On average, 1 g of fresh mass represented 0.36 g of dry mass. Values of fresh mass (t), mass at t + 7 days, and dry mass (t + 9 days) for the five gizzard contents are #1: 2.23/0.6/0.6; #2: 1.42/0.4/0.3; #3: 2.56/1.1/1.1; #4: 3.5/1.5/1.5, and #5: 2.61/1.0/1.0, respectively.

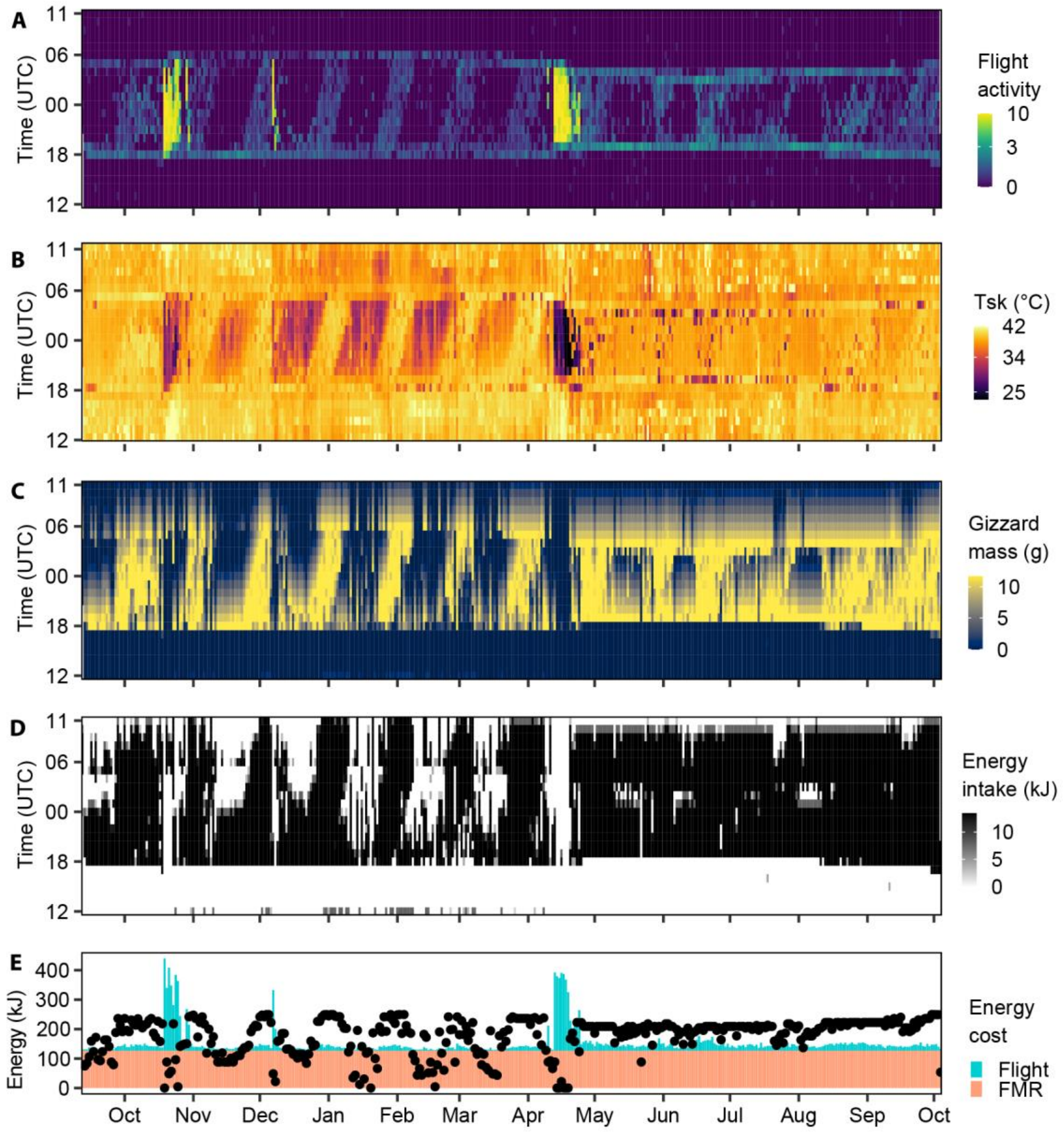

Fig. S7.

Example of output numbers from the energy model. Activity **(A)** and skin temperature **(B)** data from Fig. 1B-C. **(C)** Amount of digestive content in the gizzard per hour (empty to full: 0 - 11.72 g) based on flight activity data from current hour (A) and gizzard mass from previous hour. **(D)** Hourly metabolizable energy intake (0 - 13.4 kJ) based on a food processing rate of 1.78 g digesta (wet mass) depending on available amount of gizzard content. **(E)** Calculated daily energy intake (dots) and energy expenditure (bars) partitioned between an allometrically derived field metabolic rates (FMR) and locomotion costs (Flight).

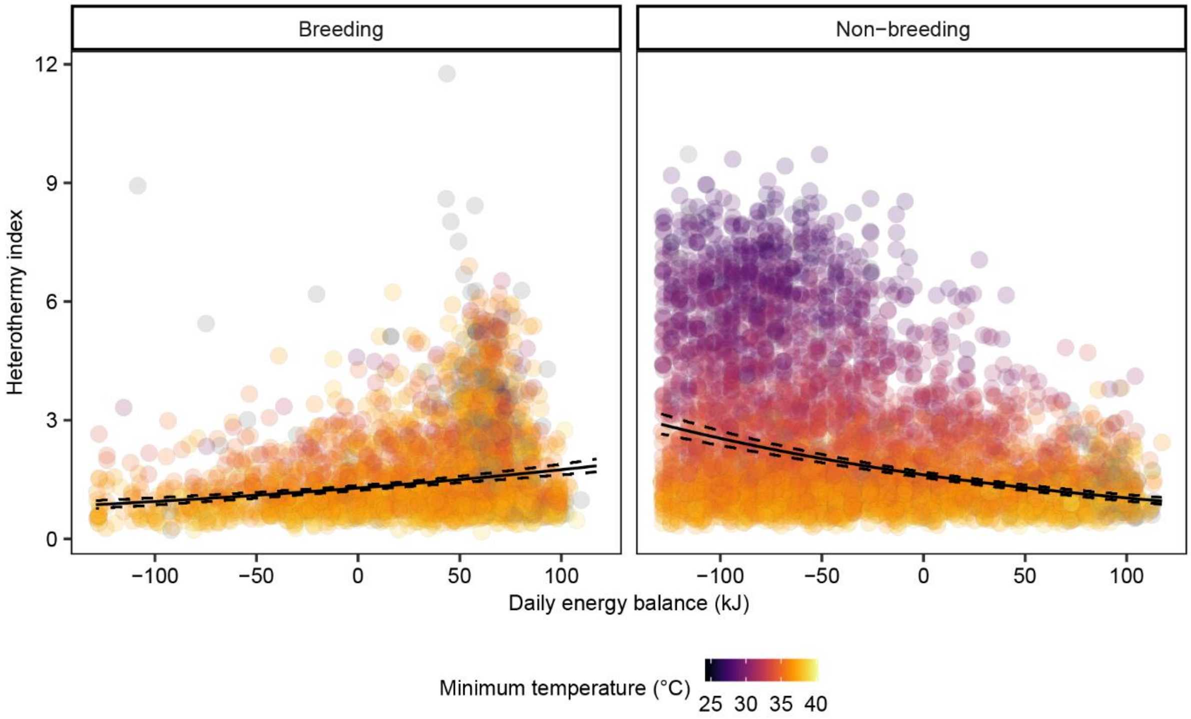

Fig. S8.

Effects of modelled daily energy balance on Heterothermy Index of red-necked nightjars in breeding and non-breeding seasons. The color of the dots reflects the nighttime minimum skin temperature. Solid and dashed lines show output from a GLMM (table S6).

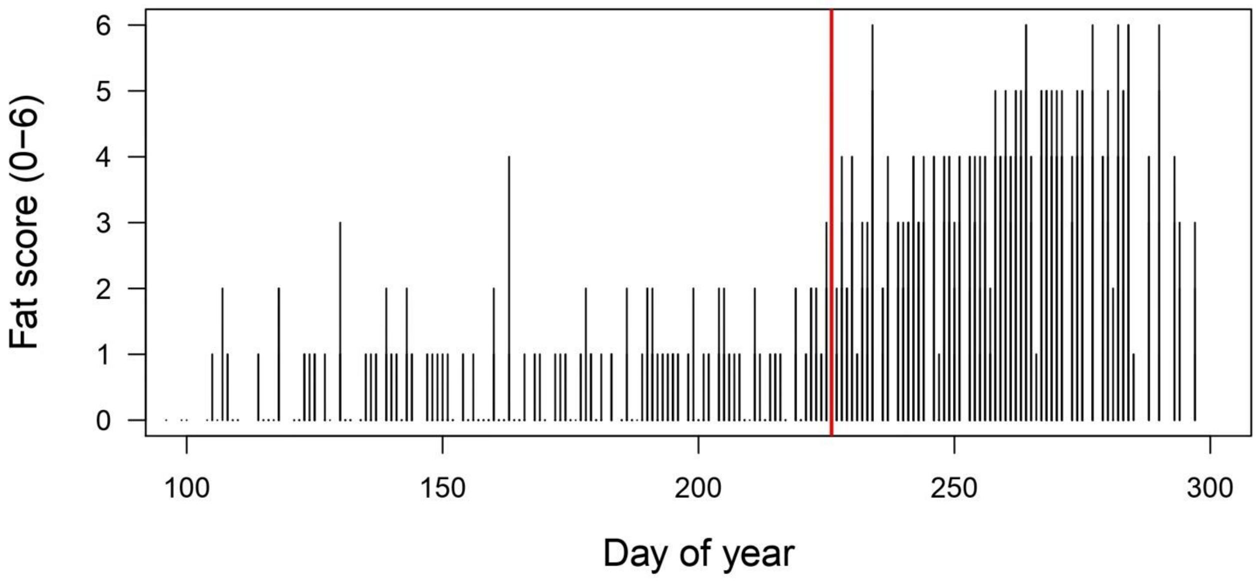

Fig. S9.

Temporal distribution of fat score records in the study population. Black vertical lines are individual fat scores. Fat scores reflect the amount of subcutaneous fat, as estimated visually according to a classification scale of 0-6. Note that fat scores >1 are rare before 15 August (day of year 227-228 depending on the year), indicated by the red vertical line, but become increasingly common after this time. Thus, the fueling period is considered to span the last ~2.5 months of the season. Days 100 and 300 correspond to 10 April and 28 October, respectively.

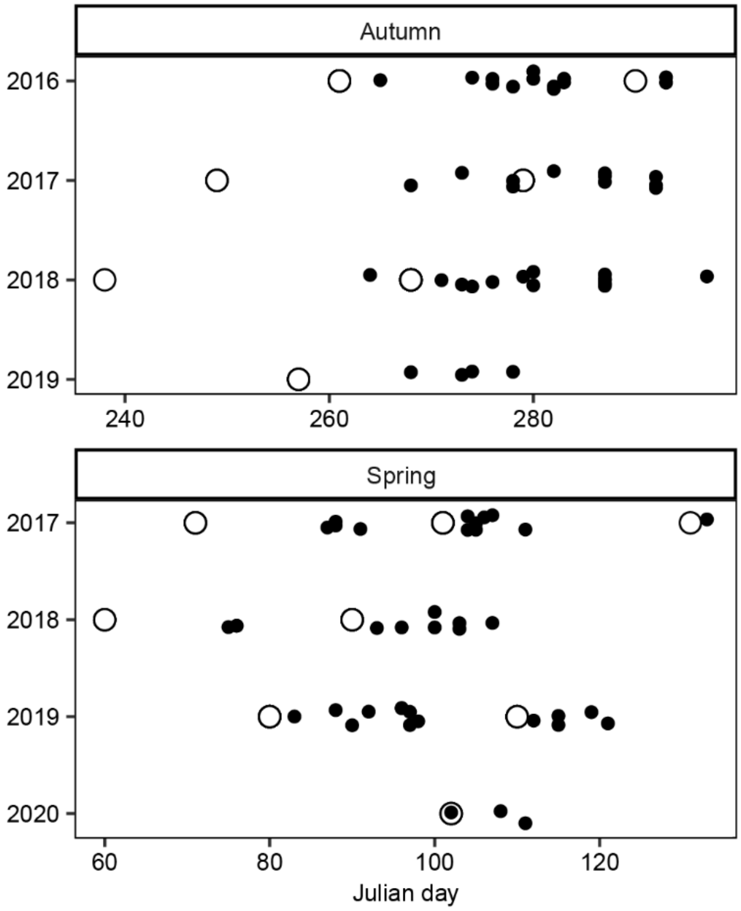

Fig. S10.

Initiation timing of the seasonal migrations relative to full moon dates. Phenological distribution of migration initiation timing in autumn (upper panel) and spring (lower panel) in four annual cycles (black dots) in relation to full-moon events (white dots). Initiation timing was on average four days later relative to the lunar cycle in autumn compared to spring.

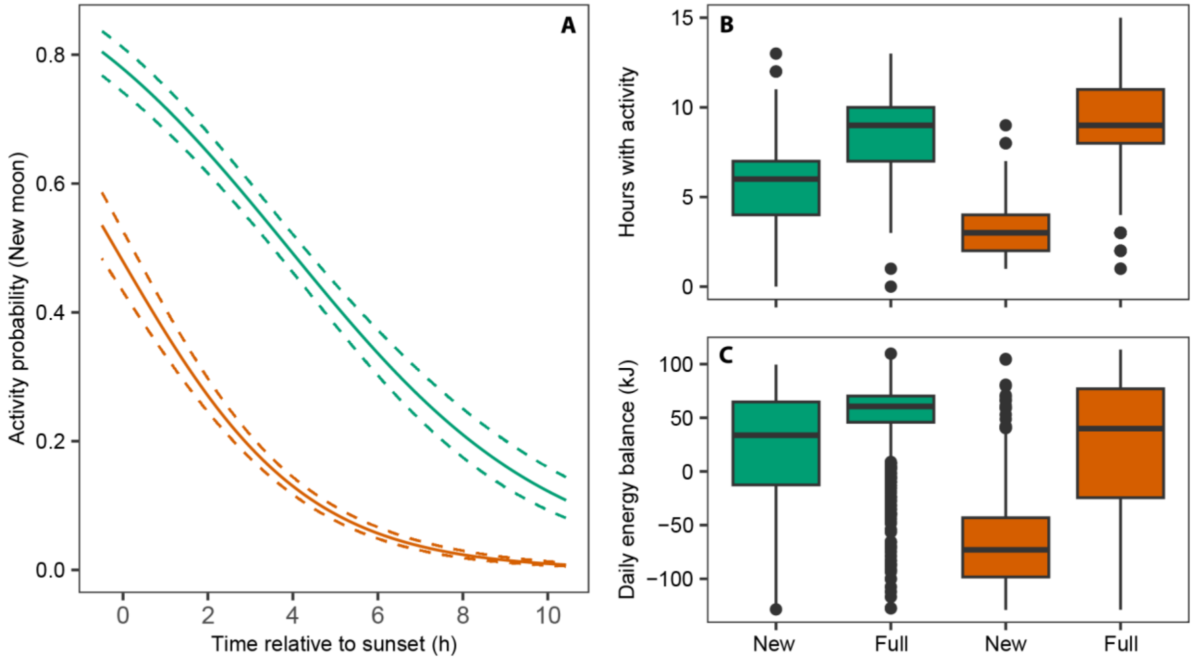

Fig. S11.

Moon phase effects on flight activity and energetics of red-necked nightjars in breeding (green) and non-breeding (orange) seasons. (A) Mean estimate of foraging activity during new moon periods in relation to time after sunset. Boxplots illustrate (B) the number of nighttime hours with activity registrations and, (C) daily energy balance during new- and full-moon phases. Notably, the lunar cycle effect on nightjar activity was more pronounced during the non-breeding season compared to the breeding season mainly due to a lower level of activity during new moon periods (A). The same pattern emerged when comparing the number of hours with activity during new moon periods in breeding nightjars (B; median = 6 hours, interquartile range (IQR) = 4 – 7) were significantly larger than during the non-breeding season (median = 3 hours, IQR = 2 – 4), Wilcoxon rank sum test: w = 69837, $P < 0.001$, $n = 29$. As a result, daily energy balance (C; median = 33.4 kJ, IQR = -13.5 – 64.4) was significantly higher during the breeding season than during the non-breeding season (median = -73.0 kJ, IQR = -98.4 – -43.1); Wilcoxon rank sum test: w = 19941, $P < 0.001$, $n = 29$.

Table S1.

Summary table of red-necked nightjars' skin temperatures recorded by MDLs during periods of nighttime activity in the breeding and non-breeding seasons. MDL XD53 failed to record temperature data from the non-breeding season and only breeding data are provided. The skin temperature data recorded by the MDLs (mean ± SD = 37.44 ± 0.64 °C) closely matched body temperatures reported for Caprimulgiformes during activity (mean ± SD = 39.7 ± 1.70 °C, n = 9 species) and resting (mean ± SD = 37.9 ± 1.51 °C, n = 7 species), respectively (*22*).

	Combined			Breeding			Non-breeding		
Tag Id	Mode	Mean	SD	Mode	Mean	SD	Mode	Mean	SD
X641	37.7	37.29	1.99	37.7	36.59	2.35	38.1	37.93	1.30
X703	37.9	37.37	1.62	37.3	37.26	1.65	37.9	37.41	1.61
X704	38.3	37.55	2.08	37.6	36.60	2.70	38.3	38.22	1.09
X705	38.0	37.54	2.03	37.3	37.00	2.29	39.0	38.28	1.27
X707	37.6	37.78	1.66	37.6	37.49	2.04	38.3	37.89	1.47
X712	39.0	38.93	1.11	38.9	38.52	1.07	39.3	39.04	1.09
X715	37.7	36.98	1.78	36.8	36.51	1.82	38.6	37.45	1.60
X721	38.5	37.96	1.45	38.8	38.38	1.30	38.3	37.75	1.47
X724	38.7	37.79	1.30	38.7	37.74	1.44	37.9	37.79	1.27
X725	37.2	36.37	2.12	36.2	35.60	2.40	37.2	37.10	1.49
X726	37.5	37.28	1.55	37.8	37.45	0.67	37.5	37.27	1.61
X727	38.3	37.90	1.85	38.8	37.48	2.29	38.3	38.29	1.22
XD33	38.7	38.09	1.63	38.4	37.75	2.00	38.7	38.36	1.18
XD35	38.2	37.33	1.81	38.7	37.31	1.92	38.2	37.34	1.77
XD36	39.2	38.49	2.02	38.3	38.18	2.32	39.4	38.88	1.47
XD39	38.0	36.95	2.10	37.0	36.60	2.68	38.0	37.12	1.74
XD42	38.6	38.02	1.75	38.6	37.75	2.15	38.6	38.20	1.37
XD49	38.2	37.63	1.55	38.1	37.00	2.00	38.2	37.87	1.26
XD52	38.7	38.23	1.45	38.7	38.18	1.37	38.8	38.28	1.52
XD53	37.7	37.01	1.30	37.7	37.01	1.30	NA	NA	NA
XD55	38.0	37.49	2.00	37.7	36.64	2.59	38.0	37.96	1.37
XD57	37.2	37.02	2.09	37.6	35.87	2.92	37.8	37.33	1.68
XD58	38.4	38.32	1.00	37.9	38.17	1.05	38.4	38.36	0.99
XC69	38.1	37.56	1.33	38.1	37.78	1.17	37.6	37.17	1.51
XC71	37.6	37.20	1.58	36.7	36.89	1.82	37.6	37.58	1.11
XC73	38.3	37.83	1.76	38.7	37.47	2.18	38.3	38.24	0.96
XC75	37.2	36.68	1.76	37.1	36.40	2.06	37.2	36.88	1.48
XC76	36.4	35.97	2.14	36.4	35.33	2.59	36.3	36.55	1.40
XC78	37.2	36.50	2.52	37.2	36.07	2.84	37.1	37.31	1.42
XC81	37.3	36.47	1.88	36.6	36.2	1.88	37.6	36.84	1.80
XC83	38.3	37.60	1.84	37.6	37.09	2.16	38.3	38.02	1.40
XC84	38.0	37.66	1.26	37.6	37.37	1.34	38.0	37.89	1.15
XC87	37.0	37.15	1.57	37.0	36.94	1.75	38.0	37.33	1.37
**Mean**	**38.0**	**37.45**	**1.72**	**37.7**	**37.11**	**1.94**	**38.09**	**37.75**	**1.39**
**SD**	**0.63**	**0.64**	**0.34**	**0.77**	**0.79**	**0.57**	**0.64**	**0.59**	**0.22**

Table S2.

Results of the GLMM examining the effect of time relative to sunset, moon phase, and their interaction on the probability of a nocturnal flight activity of red-necked nightjars. Data on nocturnal flight activity recorded by 34 MDLs of 29 individuals during the breeding season were used to model the effect of moonlight on the nocturnal flight activity of the birds on an hourly level. Random intercept and slope were included to account for variation in the detectability of activity between different MDLs. Flight activity decreases after dusk but at a lower rate during moonlit nights. Conditional R^2 = 0.28.

	Estimate	SE	z	P
Intercept	1.275	0.010	12.89	<0.001
Time relative to sunset	-0.309	0.015	-20.07	<0.001
Moon phase (Full)	0.638	0.070	9.17	<0.001
Time relative to sunset × Moon	0.196	0.014	14.12	<0.001

Random effects	Variance	SD
Intercept	0.246	0.496
Slope	0.005	0.067

Table S3.

Results of the LMM examining the effect of time relative to sunset, moon phase, and their interaction on the gizzard fullness of red-necked nightjars. Data on gizzard fullness (n = 953) recorded during 186 nights from 634 individuals during the breeding season were used to model the effect of moonlight on the foraging success of the birds. Random intercepts were included to account for repeated measures of individuals and temporal non-independence. Conditional R^2 = 0.37.

	Estimate	SE	z	P
Intercept	3.082	0.133	23.36	<0.001
Time relative to sunset	-0.175	0.029	-6.07	<0.001
Moon phase (Full)	-0.568	0.193	-2.94	<0.004
Time relative to sunset × Moon	0.160	0.041	3.86	<0.001

Random effects	Variance	SD
Intercept (Date)	0.194	0.441
Intercept (Bird Id)	0.143	0.378

Table S4.

Birds captured ≥2 times on the same night and used to estimate the rate of mass loss, assumed to represent defecation rate and, therefore, the rate of food processing (FPR) of adult red-necked nightjars under field conditions. FPR presented here are derived from recorded defecation rate assuming an absorption fraction (f_a) of 0.113 (*12*).

Bird ID	Sex	Capture date	Mass loss (g)	Time elapsed (h)	FPR (g × h^{-1})
1B45863*	Female	30/6/20	-9.70	2.75	3.96
3149789	Male	26/5/16	-2.60	1.22	2.40
1B28113	Male	19/9/19	-1.70	1.00	1.91
3222649	Male	27/8/10	-3.00	2.13	1.58
3222663	Female	31/8/10	-2.00	1.43	1.57
3384119	Female	24/7/15	-0.70	0.55	1.43
3260945	Female	10/5/11	-4.00	3.43	1.31
3260997	Female	23/5/14	-0.50	0.43	1.30
3222668	Female	5/9/10	-6.00	6.38	1.06
1B23709	Male	8/7/16	-2.60	4.25	0.69
1B28187	Male	21/6/19	-1.10	1.95	0.63
3222638	Female	20/8/10	-1.10	2.05	0.60
3149750	Female	11/7/13	-1.80	4.00	0.51
3149767	Female	10/6/13	-1.60	3.58	0.50
3384144	Female	17/7/14	-1.20	2.85	0.47
1B09555	Female	13/7/16	-1.40	3.33	0.47
1B09505	Female	21/5/15	-1.20	3.50	0.39
3260906	Male	6/6/12	-0.40	1.33	0.34
1B45875	Female	31/7/20	-0.10	0.33	0.34
Mean**					1.78
SD**					0.39

* Not considered in the analysis. FPR of this bird almost doubles the next highest rate and is therefore considered a measurement error.

** For the top five individuals, used in the subsequent analyses.

Table S5.

Results of the GLMM examining the effect of daily energy balance (in kJ), season (breeding *vs.* non-breeding), and their interaction on the probability of entering torpor in red-necked nightjars. Data on the occurrence of nighttime torpor were available from 7,813 nights recorded by 33 MDLs on 28 individuals. Random intercept and slope were included to account for variation in the detectability of activity between different MDLs. Conditional $R^2 = 0.42$.

	Estimate	SE	z	P
Intercept	-2.876	0.151	-19.03	<0.001
Energy balance (kJ)	-0.002	0.002	-1.38	0.17
Season (Non-breeding)	1.897	0.105	18.07	<0.001
Energy balance × Season	-0.012	0.002	-6.84	<0.001

Random effects	Variance	SD
Intercept	0.448	0.669
Slope	0.000	0.003

Table S6.

Results of the LMM examining the effect of daily energy balance, season, and their interaction on the daily Heterothermy Index (log10 transformed) of red-necked nightjars. Data on the occurrence of nighttime torpor were available from 7,813 nights recorded by 33 MDLs on 28 individuals. Random intercept and slope were included to account for variation in the detectability of activity between different MDLs. Conditional $R^2 = 0.19$.

	Estimate	SE	z	P
Intercept	0.110	0.011	10.24	<0.001
Daily energy balance (kJ)	0.001	0.000	8.26	<0.001
Season (Non-breeding)	0.101	0.008	12.80	<0.001
DEB × Season	-0.003	0.000	-25.84	<0.001

Random effect	Variance	SD
Intercept	0.002	0.045
Slope	0.000	0.001

Table S7.

Results of the GLMM examining the effect of the lunar cycle on the probability of entering torpor during the non-breeding season in red-necked nightjars. Data on the occurrence of nighttime torpor were available from 7,813 nights recorded by 33 MDLs on 28 individuals. Random intercept was included to account for individual variation. Conditional $R^2 = 0.27$.

	Estimate	SE	Z	P
Intercept	-2.334	0.147	-15.91	<0.001
Lunar cycle (+14 days)	2.260	0.087	26.05	<0.001

Random effects	Variance	SD
Intercept	0.584	0.764

Table S8.

Results of the LMM examining the temporal correlation between moonset time and initiation of torpor in individual nightjars across the annual cycle. Random intercept was included to account for individual variation. 760 days with nighttime moonsets and 28 individuals. Conditional R^2 = 0.41.

	Estimate	SE	z	P
Intercept	4.191	0.352	11.89	<0.001
Moonset	0.659	0.030	22.02	<0.001

Random effects	Variance	SD
Intercept	0.123	0.350

Table S9.

Results of the LMM examining the effect of the lunar cycle on body mass (corrected for gizzard score) in red-necked nightjars (997 observations of 661 individuals across 10 seasons). Random intercepts were included to account for systematic differences between individuals and years, respectively. Conditional R^2 = 0.26.

	Estimate	SE	z	P
Intercept	90.01	0.603	149.27	<0.001
Lunar cycle (-6)	5.264	0.788	6.68	<0.001

Random effects	Variance	SD
Bird Id	18.111	4.256
Year	0.998	0.999

Table S10.

Results of a LMM examining the effect of the lunar cycle on fuel deposition rates in red-necked nightjars (52 repeated observations of 52 individuals across 10 seasons). Mass change was included as a continuous dependent variable and time elapsed between measurements (days) and the average number of hours with the moon above the horizon were included as main predictors. Random intercept was included to account for systematic difference between years. Conditional $R^2 = 0.33$.

	Estimate	SE	z	P
Intercept	-13.08	3.203	-4.08	<0.001
Duration (days)	0.432	0.144	2.99	0.003
Moonlight (hours)	1.438	0.553	2.60	0.009

Random effects	Variance	SD
Year	2.469	1.571

Table S11.

Results of the GLMM examining the effect of the lunar cycle on the probability of daily migratory flights of tracked nightjars in spring. Random intercepts (bird identity *n* = 29 and year *n* = 4) were included to account for repeated measurements of the same individuals, and systematic differences between different breeding seasons. Conditional R^2 = 0.07.

	Estimate	SE	z	P
Intercept	-2.323	0.118	-19.77	<0.001
Lunar cycle (+14 days)	1.347	0.152	8.84	<0.001

Random effects	Variance	SD
Intercept (Bird Id)	0.007	0.085
Intercept (Year)	0.007	0.086

Table S12.

Results of the GLMM examining the effect of the lunar cycle on the probability of daily migratory flights of tracked nightjars in autumn. Random intercepts (bird identity *n* = 30 and year *n* = 4) were included to account for repeated measurements of the same individuals, and systematic differences between different breeding seasons. Conditional $R^2 = 0.04$.

	Estimate	SE	z	P
Intercept	-1.857	0.101	-18.39	<0.001
Lunar cycle (-8 days)	0.873	0.145	6.03	<0.001

Random effects	Variance	SD
Intercept (Bird Id)	0.029	0.171
Intercept (Year)	0.000	0.000

Table S13.

Results of a LMM examining the seasonal effect on the initiation timing of autumn and spring migrations in relation to the timing of the preceding full moon. Random intercept was included to account for repeated measurement within each track. n = 82 observations from 43 annual cycles. Conditional R^2 = 0.13.

	Estimate	SE	z	P
Intercept	15.163	1.003	15.11	<0.001
Relative timing (days)	-5.163	1.455	-3.55	<0.001

Random effects	Variance	SD
Track Id	0.000	0.002

Table S14.

Results from a multi-modal analysis of the number of trapped incubating female red-necked nightjars. Numbers correspond to days since the first full moon in May for each year (2011-2020). Peak and nadir refer to the timing modes and anti-modes and the corresponding density estimates, using a critical bandwidth of 5.25.

Full moon	New moon	Peak	Nadir	$Density_{Peak}$	$Density_{Nadir}$
30	16	16	25	0.010	0.008
60	45	44	59	0.016	0.010
89	75	68	NA	0.013	NA

Table S15.

Results of the GLMM examining the effect of the lunar cycle on the probability of catching an incubating female red-necked nightjar (967 observations of 525 individuals across 10 seasons). Random intercepts (bird identity and year) were included to account for repeated measurements of the same individuals, and systematic differences between different breeding seasons. Conditional R^2 = 0.06.

	Estimate	SE	z	P
Intercept	-0.782	0.193	-4.06	<0.001
Lunar cycle (-10 days)	0.460	0.197	2.33	0.02

Random effects	Variance	SD
Intercept (Bird Id)	0.002	0.044
Intercept (Year)	0.191	0.437

Table S16.

Summary table of minimum daily translocation distance (in meters) on breeding and non-breeding sites for five red-necked nightjars equipped with GPS tags. The minimum daily translocation distance in the breeding season was on average 2171 meters, (SD = 3116, n = 525), which was significantly longer than in the non-breeding season (mean = 327, SD = 521, n = 426, LMM = 1844.7, SE = 152.5, z = 12.10, $P < 0.001$, n = 951, groups = 5).

	Breeding			Non-breeding		
Bird Id	Mean	SD	n	Mean	SD	n
T20421	2244	200	125	465	815	81
T20518	2980	4760	152	281	327	99
T20522	1516	1703	131	426	597	106
T20523	1691	2355	77	202	247	71
T20525	1942	2237	40	204	279	69

Table S17.
Measurements of adult red-necked nightjars and output from the *afpt* R-package.

Bird ID	Wingspan (m)	Wing area (m^2)	Aspect ratio	U_m ($m\ s^{-1}$)	Pch_{Um} (kJ)
3142699	0.5557	0.0501	6.16	9.15	4.19
3149764	0.5866	0.0507	6.79	8.93	4.02
3218577	0.6165	0.0508	7.48	8.76	3.88
3218583	0.6072	0.0508	7.25	8.81	3.92
3218584	0.5831	0.0491	6.92	9	4.05
3222369	0.6163	0.0535	7.11	8.69	3.86
3288623	0.6000	0.0512	7.03	8.84	3.95
3377679	0.5842	0.0482	7.08	9.02	4.06
3384116	0.5917	0.0499	7.02	8.93	4
3384119	0.5736	0.0493	6.68	9.05	4.1
3384123	0.6054	0.0518	7.07	8.79	3.92
3384138	0.5974	0.0541	6.6	8.78	3.94
3384212	0.6139	0.0514	7.33	8.76	3.89
3396928	0.6038	0.0530	6.88	8.77	3.92
1B09503	0.5878	0.0519	6.66	8.89	4.01
1B09505	0.5921	0.0479	7.32	8.98	4.02
1B09508	0.6080	0.0515	7.18	8.79	3.91
1B09700	0.6023	0.0507	7.16	8.84	3.95
1B23566	0.5941	0.0489	7.26	8.95	4.01
1B24312	0.5634	0.0482	6.58	9.15	4.16
1B24329	0.5912	0.0480	7.29	8.99	4.03
1B24337	0.5878	0.0493	7.01	8.97	4.03
1B28432	0.6029	0.0526	6.91	8.79	3.92
1B41624	0.5897	0.0489	7.1	8.97	4.02
1B57610	0.5832	0.0494	6.89	8.99	4.05
**Mean**	**0.5935**	**0.0543**	**6.99**	**8.9**	**3.99**

REFERENCES

1. J. R. Bernhardt, M. I. O'Connor, J. M. Sunday, A. Gonzalez, Life in fluctuating environments. *Philos. Trans. R. Soc. Lond. B Biol. Sci.* **375**, 20190454 (2020).

2. S. Åkesson, B. Helm, Endogenous programs and flexibility in bird migration. *Front. Ecol. Evol.* **8**, 78 (2020).

3. R. A. Hut, D. G. Beersma, Evolution of time-keeping mechanisms: Early emergence and adaptation to photoperiod. *Philos. Trans. R. Soc. Lond. B Biol. Sci.* **366**, 2141–2154 (2011).

4. T. Alerstam, A. Hedenström, S. Åkesson, Long-distance migration: Evolution and determinants. *Oikos* **103**, 247–260 (2003).

5. J. B. Armstrong, D. E. Schindler, Excess digestive capacity in predators reflects a life of feast and famine. *Nature* **476**, 84–87 (2011).

6. T. Piersma, J. A. van Gils, *The Flexible Phenotype: A Body-Centred Integration of Ecology, Physiology, and Behaviour* (Oxford Univ. Press, 2011).

7. R. M. Brigham, R. M. Barclay, Lunar influence on foraging and nesting activity of common poorwills (*Phalaenoptilus nuttallii*). *Auk* **109**, 315–320 (1992).

8. B. Smit, J. G. Boyles, R. M. Brigham, A. E. McKechnie, Torpor in dark times: Patterns of heterothermy are associated with the lunar cycle in a nocturnal bird. *J. Biol. Rhythms* **26**, 241–248 (2011).

9. F. Broekhuis, S. Grünewälder, J. W. McNutt, D. W. Macdonald, Optimal hunting conditions drive circalunar behavior of a diurnal carnivore. *Behav. Ecol.* **25**, 1268–1275 (2014).

10. P. Pinet, A. Jaeger, E. Cordier, G. Potin, M. Le Corre, Celestial moderation of tropical seabird behavior. *PLOS ONE* **6**, e27663 (2011).

11. A. Hedenström, R. A. Sparks, G. Norevik, C. Woolley, G. J. Levandoski, S. Åkesson, Moonlight drives nocturnal vertical flight dynamics in black swifts. *Curr. Biol.* **32**, 1875–1881.e3 (2022).

12. K. S. Last, L. Hobbs, J. Berge, A. S. Brierley, F. Cottier, Moonlight drives ocean-scale mass vertical migration of zooplankton during the Arctic winter. *Curr. Biol.* **26**, 244–251 (2016).

13. K. J. Gaston, Nighttime ecology: The "nocturnal problem" revisited. *Am. Nat.* **193**, 481–502 (2019).

14. D. T. Holyoak, *Nightjars and Their Allies: The Caprimulgiformes* (Oxford Univ. Press, 2001).

15. W. Jetz, J. Steffen, K. E. Linsenmair, Effects of light and prey availability on nocturnal, lunar and seasonal activity of tropical nightjars. *Oikos* **103**, 627–639 (2003).

16. G. Norevik, S. Åkesson, A. Andersson, J. Bäckman, A. Hedenström, The lunar cycle drives migration of a nocturnal bird. *PLOS Biol.* **17**, e3000456 (2019).

17. R. Evens, C. Kowalczyk, G. Norevik, E. Ulenaers, B. Davaasuren, S. Bayargur, T. Artois, S. Åkesson, A. Hedenström, F. Liechti, M. Valcu, B. Kempenaers, Lunar synchronization of daily activity patterns in a crepuscular avian insectivore. *Ecol. Evol.* **10**, 7106–7116 (2020).

18. H. D. Jackson, The food of the Afrotropical nightjars. *Ostrich* **71**, 408–415 (2000).

19. H. D. Jackson, A review of foraging and feeding behaviour and associated anatomical adaptations in Afrotropical nightjars. *Ostrich* **74**, 187–204 (2003).

20. R. M. Brigham, Daily torpor in a free-ranging goatsucker, the common poorwill (*Phalaenoptilus nuttallii*). *Physiol. Zool.* **65**, 457–472 (1992).

21. J. E. Lane, D. L. Swanson, R. M. Brigham, A. E. McKechnie, Physiological responses to temperature by whip-poor-wills: More evidence for the evolution of low metabolic rates in Caprimulgiformes. *Condor* **106**, 921–925 (2004).

22. R. Prinzinger, A. Preßmar, E. Schleucher, Body temperature in birds. *Comp. Biochem. Physiol. A Physiol.* **99**, 499–506 (1991).

23. A. B. Lang, E. K. Kalko, H. Römer, C. Bockholdt, D. K. Dechmann, Activity levels of bats and katydids in relation to the lunar cycle. *Oecologia* **146**, 659–666 (2006).

24. H. D. Jackson, A field survey to investigate why nightjars frequent roads at night. *Ostrich* **74**, 97–101 (2003).

25. P. A. Bednekoff, A. I. Houston, Avian daily foraging patterns: Effects of digestive constraints and variability. *Evol. Ecol.* **8**, 36–52 (1994).

26. R. Macleod, P. Barnett, J. A. Clark, W. Cresswell, Body mass change strategies in blackbirds *Turdus merula*: The starvation–predation risk trade-off. *J. Anim. Ecol.* **74**, 292–302 (2005).

27. E. R. Price, A. Brun, E. Caviedes-Vidal, W. H. Karasov, Digestive adaptations of aerial lifestyles. *Physiology* **30**, 69–78 (2015).

28. J. A. van Gils, T. Piersma, A. Dekinga, M. W. Dietz, Cost–benefit analysis of mollusc-eating in a shorebird II: Optimizing gizzard size in the face of seasonal demands. *J. Exp. Biol.* **206**, 3369–3380 (2003).

29. M. Kersten, W. Visser, The rate of food processing in the oystercatcher: Food intake and energy expenditure constrained by a digestive bottleneck. *Funct. Ecol.* **10**, 440–448 (1996).

30. A. M. Mills, Latitudinal gradients of biologically useful semi-darkness. *Ecography* **31**, 578–582 (2008).

31. D. W. Thomas, R. M. Brigham, H. Lapierre, Field metabolic rates and body mass changes in common poorwills (*Phalaenoptilus nuttallii*). *Écoscience* **3**, 70–74 (1996).

32. L. Jenni, S. Jenni-Eiermann, Fuel supply and metabolic constraints in migrating birds. *J. Avian Biol.* **29**, 521–528 (1998).

33. Å. Lindström, "Fuel deposition rates in migrating birds: Causes, constraints and consequences," in *Avian Migration* (Springer-Verlag, 2003), pp. 307–320.

34. A. M. Korpach, C. M. Davy, A. M. Mills, K. C. Fraser, Lunar synchrony, geography and individual clocks shape autumn migration timing in an avian migrant. *Behav. Ecol.* **35**, arae001 (2024).

35. A. M. Mills, The influence of moonlight on the behavior of goatsuckers (Caprimulgidae). *Auk* **103**, 370–378 (1986).

36. P. A. English, J. J. Nocera, D. J. Green, Nightjars may adjust breeding phenology to compensate for mismatches between moths and moonlight. *Ecol. Evol.* **8**, 5515–5529 (2018).

37. C. M. Perrins, H. Q. P. Crick, Influence of lunar cycle on laying dates of European nightjars (*Caprimulgus europaeus*). *Auk* **113**, 705–708 (1996).

38. Å. Lindström, H. G. Visser, S. Daan, The energetic cost of feather synthesis is proportional to basal metabolic rate. *Physiol. Zool.* **66**, 490–510 (1993).

39. C. Camacho, Tropical phenology in temperate regions: Extended breeding season in a long-distance migrant. *Condor* **115**, 830–837 (2013).

40. R. Evens, M. Lathouwers, J. N. Pradervand, A. Jechow, C. C. M. Kyba, T. Shatwell, A. Jacot, E. Ulenaers, B. Kempenaers, M. Eens, Skyglow relieves a crepuscular bird from visual constraints on being active. *Sci. Total Environ.* **900**, 165760 (2023).

41. C. A. Adams, C. C. St. Clair, E. C. Knight, E. M. Bayne, Behaviour and landscape contexts determine the effects of artificial light on two crepuscular bird species. *Landsc. Ecol.* **39**, 83 (2024).

42. E. Malmqvist, S. Jansson, S. Zhu, W. Li, K. Svanberg, S. Svanberg, J. Rydell, Z. Song, J. Bood, M. Brydegaard, S. Åkesson, The bat–bird–bug battle: Daily flight activity of insects and their predators over a rice field revealed by high-resolution Scheimpflug Lidar. *R. Soc. Open Sci.* **5**, 172303 (2018).

43. K. M. Gaynor, C. E. Hojnowski, N. H. Carter, J. S. Brashares, The influence of human disturbance on wildlife nocturnality. *Science* **360**, 1232–1235 (2018).

44. O. Levy, T. Dayan, W. P. Porter, N. Kronfeld-Schor, Time and ecological resilience: Can diurnal animals compensate for climate change by shifting to nocturnal activity? *Ecol. Monogr.* **89**, e01334 (2019).

45. C. Camacho, S. Palacios, P. Sáez, S. Sánchez, J. Potti, Human-induced changes in landscape configuration influence individual movement routines: Lessons from a versatile, highly mobile species. *PLOS ONE* **9**, e104974 (2014).

46. G. Norevik, S. Åkesson, A. Hedenström, The spatial consistency and repeatability of migratory flight routes and stationary sites of individual European nightjars based on multiannual GPS tracks. *Mov. Ecol.* **13**, 8 (2025).

47. R. Evens, N. Beenaerts, T. Neyens, N. Witters, K. Smeets, T. Artois, Proximity of breeding and foraging areas affects foraging effort of a crepuscular insectivorous bird. *Sci. Rep.* **8**, 3008 (2018).

48. Y. Wasserlauf, A. Gancz, A. Ben Dov, R. Efrat, N. Sapir, R. Dor, O. Spiegel, A telemetry study shows that an endangered nocturnal avian species roosts in extremely dry habitats to avoid predation. *Sci. Rep.* **13**, 11888 (2023).

49. V. Agafonkin, B. Thieurmel, "suncalc: Compute sun position, sunlight phases, moon position, and lunar phase," R package version 0.4 (2018).

50. B. Lundgren, A. Hedenström, J. Pettersson, Correlation between body components and visible fat index in the willow warbler *Phylloscopus trochilus*. *Ornis Svec.* **5**, 75–79 (1995).

51. R. Wirestam, T. Fagerlund, M. Rosén, A. Hedenström, Magnetic resonance imaging for noninvasive analysis of fat storage in migratory birds. *Auk* **125**, 965–971 (2008).

52. J. A. McLean, J. R. Speakman, Energy budgets of lactating and non-reproductive brown long-eared bats (*Plecotus auritus*). *Funct. Ecol.* **13**, 360–372 (1999).

53. W. H. Karasov, Digestion in birds: Chemical and physiological determinants and ecological implications. *Stud. Avian Biol.* **13**, 1–4 (1990).

54. C. J. Whelan, J. S. Brown, Optimal foraging and gut constraints: Reconciling two schools of thought. *Oikos* **110**, 481–496 (2005).

55. K. A. Nagy, I. A. Girard, T. K. Brown, Energetics of free-ranging mammals, reptiles, and birds. *Annu. Rev. Nutr.* **19**, 247–277 (1999).

56. L. I. Doucette, F. Geiser, Seasonal variation in thermal energetics of the Australian owlet-nightjar (*Aegotheles cristatus*). *Comp. Biochem. Physiol. A Mol. Integr. Physiol.* **151**, 615–620 (2008).

57. R. N. Hardy, *Temperature and Animal Life* (Edward Arnold, London, 1972).

58. K. A. Nagy, Field metabolic rate and food requirement scaling in mammals and birds. *Ecological monographs* **57**, 111–128 (1987).

59. M. Klein Heerenbrink, L. C. Johansson, A. Hedenström, Power of the wingbeat: Modelling the effects of flapping wings in vertebrate flight. *Proc. Math. Phys. Eng. Sci.* **471**, 20140952 (2015).

60. C. J. Pennycuick, *Modelling the Flying Bird* (Academic Press, 2008).

61. B. Bruderer, A. Boldt, Flight characteristics of birds: I. Radar measurements of speeds. *Ibis* **143**, 178–204 (2001).

62. C. Camacho, 'Bodyguard' plants: Predator-escape performance influences microhabitat choice by nightjars. *Behav. Processes* **103**, 145–149 (2014).

63. J. G. Boyles, B. Smit, A. E. McKechnie, A new comparative metric for estimating heterothermy in endotherms. *Physiol. Biochem. Zool.* **84**, 115–123 (2011).

64. V. M. Muggeo, Segmented: An R package to fit regression models with broken-line relationships. *R News* **8**, 20–25 (2008).

65. T. Alerstam, Å. Lindström, “Optimal bird migration: The relative importance of time, energy and safety,” in *Bird Migration: Physiology and Ecophysiology* (Springer, 1990), pp. 331–351.

66. Å. Lindström, T. Alerstam, Optimal fat loads in migrating birds: A test of the time-minimization hypothesis. *Am. Nat.* **140**, 477–491 (1992).

67. M. E. Brooks, B. M. Bolker, K. Kristensen, M. Maechler, “glmmTMB: Generalized linear mixed models using Template Model Builder,” R package version 1 (2022).

68. F. Hartig, “DHARMa: Residual diagnostics for hierarchical regression models,” R package version 0.3.3 (2020).

69. S. Nakagawa, H. Schielzeth, A general and simple method for obtaining R^2 from generalized linear mixed-effects models. *Methods Ecol. Evol.* **4**, 133–142 (2013).

70. K. Barton, M. K. Barton, “MuMIn*:* Multi-Model Inference,” R package version 1 (2019).

71. M. Ameijeiras-Alonso, R. M. Crujeiras, A. Rodríguez-Casal, multimode: An R package for mode assessment. *J. Stat. Softw.* **97**, 1–32 (2021).

72. M. K. Śmielak, Biologically meaningful moonlight measures and their application in ecological research. *Behav. Ecol. Sociobiol.* **77**, 21 (2023).

73. C. Camacho, Early age at first breeding and high natal philopatry in the red-necked nightjar *Caprimulgus ruficollis*. *Ibis* **156**, 442–445 (2014).

74. P. Hidalgo-Rodríguez, P. Sáez-Gómez, J. Blas, A. Hedenström, C. Camacho, Body mass dynamics of migratory nightjars are explained by individual turnover and fueling. *Behav. Ecol.* **32**, 1086–1093 (2021).

75. P. Sáez-Gómez, S. Palacios, C. Camacho, Landscape change promotes the emergence of a rare predator–prey interaction. *Food Webs* **15**, e00078 (2018).

76. G. Gargallo, Flight feather moult in the red-necked nightjar *Caprimulgus ruficollis*. *J. Avian Biol.* **25**, 119–124 (1994).
